# Boson peak in covalent network glasses: Isostaticity and marginal stability

**DOI:** 10.1073/pnas.2528998123

**Published:** 2026-05-29

**Authors:** Hideyuki Mizuno, Tatsuya Mori, Giacomo Baldi, Emi Minamitani

**Affiliations:** ^a^ Graduate School of Arts and Sciences, The University of Tokyo, Tokyo 153-8902, Japan; ^b^ Department of Materials Science, University of Tsukuba, Ibaraki 305-8573, Japan; ^c^ Department of Physics, University of Trento, Povo (Tn) 38123, Italy; ^d^ The Institute of Scientific and Industrial Research, The University of Osaka, Osaka 567-0047, Japan

**Keywords:** amorphous materials, covalent network glasses, boson peak, isostaticity, marginal stability

## Abstract

The excess vibrational states relative to the Debye model of solids, referred to as the boson peak (BP), are a key feature of glasses and amorphous materials. These excess states underlie anomalous thermal properties such as excess specific heat and low thermal conductivity, as well as mechanical characteristics such as nonaffine elasticity and brittle plasticity. Despite its importance, understanding of the BP remains limited in covalent network glasses. The most promising concepts are isostaticity and marginal stability, which have been established in theories of rigidity percolation and the jamming transition. While these concepts, supported by extensive data, account for the BP in packing-based glasses, comparable explanations have not yet been demonstrated for covalent network glasses. Here we study silica glass, a prototypical covalent network glass, using molecular dynamics simulations. We show that the BP in silica glass is governed by near-isostatic constraints and marginal stability, supporting the universality of these concepts across diverse glassy systems. Furthermore, we reveal that these principles manifest as a wavenumber-independent band in the dynamical structure factor, and we demonstrate agreement with inelastic X-ray scattering data. Our results provide an experimentally testable framework for deciphering the BP and for refining the interpretation of scattering data in amorphous materials.

## Significance

Excess nonphononic excitations beyond the Debye theory of phonons are central to understanding amorphous materials. Extensive light, inelastic X-ray, and neutron scattering experiments have documented these excitations in covalent network glasses, yet theory has largely focused on simple packing-based glasses and only rarely addressed experimentally measured dynamical structure factors. Here, we study silica glass and explain its nonphononic excitations and dynamical structure factor using isostaticity and marginal stability, principles established in rigidity percolation and jamming. We show that these principles generate a wavenumber-independent band in the dynamical structure factor and demonstrate agreement with inelastic X-ray scattering data for silica glass. Our findings bridge the gap between theory and experiment and provide practical criteria for interpreting scattering measurements

The vibrational states of atoms and molecules in crystals are well characterized as phonons, providing a clear understanding of their material properties (1). In contrast, the vibrational states in glasses deviate markedly from phonons, producing anomalous properties relative to crystals (2). Whereas the vibrational density of states (vDOS) in crystals is accurately captured by Debye theory based on phonons, glasses exhibit an excess over the Debye prediction, an anomaly known as the boson peak (BP) (2, 3). The BP has been observed in dynamical structure factors measured by light, inelastic X-ray, and neutron scattering, establishing it as an experimental hallmark of glasses (4–18). The excess vibrational states over the Debye prediction are directly linked to the excess specific heat of glasses and strongly influence transport properties including sound propagation and heat conduction (19–24). They also affect nonaffine elastic responses and plastic deformation in glasses (25–28). Thus, the BP is central to understanding glasses and amorphous materials.

Understanding of the BP has advanced over decades. Several theoretical frameworks have been proposed, including elastic heterogeneities (29–33), soft-potential models (34–37), random-matrix approaches (38, 39), and mode-coupling-based approaches (40). Among these, early works (41–44) introduced the concept of isostaticity based on the topology of random networks. They accounted for the rigidity of covalent network glasses by counting constraints (bonds) against degrees of freedom. When the number of constraints falls below the number of degrees of freedom, the system can deform continuously at no energy cost, and zero-energy vibrational modes, known as floppy modes, appear. Conversely, when constraints exceed degrees of freedom, excess constraints render energy-free deformations costly, shifting floppy modes to finite frequencies. The boundary where constraints equal degrees of freedom defines the isostatic state, the rigidity threshold known as Maxwell’s criterion (45). Thus, by tracking how constraints surpass degrees of freedom, one can assess rigidity and the nature of low-frequency vibrational states. This concept was developed within the framework of rigidity percolation (46–49) and has also been employed to explain low-energy dynamics and two-level tunneling states in glasses (50, 51).

Subsequently, isostaticity was incorporated into the physics of jammed materials, providing insights into the jamming and glass transitions (52–54). In this context, the topological picture described above was unified with energetic considerations through the notion of internal stress or prestress, which arise from frustration in amorphous systems (55–57). Dense and disordered packings generate strong frustration, leading to internal stresses that originate microscopically from repulsive interparticle forces. It has been proposed that glasses are solids that are marginally stable against these frustration-induced internal stresses. From the vibrational perspective, internal stresses shift modes that would be floppy at isostaticity but are pushed to finite frequencies by excess constraints back toward low frequencies. This produces a gapless vDOS that extends down to zero frequency and signals marginal stability. These modes contribute to the excess low-frequency vDOS and form the BP. Consequently, the BP can be viewed as a manifestation of isostaticity and marginal stability. Building upon this scenario, a mean-field theory was constructed using random spring networks, which successfully explains the BP (58, 59).

Numerical simulations have tested this scenario and its mean-field predictions. In particular, the packing model of harmonic spheres (HS) has been extensively studied in the context of the jamming transition (52–54). Jamming scaling laws for the vDOS and the BP have been established (60–65), consistent with principles of isostaticity and marginal stability (55–57) and with mean-field theory (58, 59). Isostaticity and marginal stability have also been confirmed for the BP of Lennard–Jones (LJ) glass, a representative model of van der Waals glasses (66–68), and hard-sphere glass, a representative model of colloidal glasses (69, 70). Across these simulations, quasi-localized vibrations (QLVs) emerge alongside the BP as a consequence of marginal stability driven by frustration-induced internal stresses (61, 63, 71, 72). We note that, in the present work, we define the system to be in a marginally stable state when the vDOS of QLVs is gapless. Thus, the BP is now well rationalized in packing-based glasses, including particle packings, van der Waals glasses, and colloidal glasses, in terms of isostaticity and marginal stability.

In contrast, the BP remains less well understood in network-forming glasses, that is, covalent network glasses such as oxide glasses. Extensive light, inelastic X-ray, and neutron scattering experiments have measured the dynamical structure factor of covalent network glasses and repeatedly observed the BP (4, 6–8, 10, 11, 13, 14, 16–18). The concept of isostaticity was originally proposed to interpret these experimental obser- vations in covalent network glasses (41–44). However, a detailed validation of isostaticity, analogous to that achieved for packing, van der Waals, and colloidal glasses, has not yet been performed. Moreover, marginal stability driven by internal stresses remains largely unexplored in this class of materials. In addition, how isostaticity and marginal stability manifest in the experimentally measured dynamical structure factor has not been clarified. Given the breadth of scattering data on covalent network glasses, establishing the BP mechanism in these materials and its expression in the dynamical structure factor is a crucial step toward bridging theoretical frameworks and experiments.

In this work, we focus on silica (${\text{SiO}}_{2}$) glass, a representative oxide glass, to investigate its BP. Previous numerical studies have examined the vDOS and QLVs in silica glass (73–76). In contrast to these studies, our primary goal is to understand the BP in terms of isostaticity and marginal stability. We further analyze the dynamical structure factor to clarify how isostaticity and marginal stability are expressed in this experimentally accessible observable. We find that the BP in silica glass follows from near-isostatic constraints and marginal stability, as in HS and LJ glasses, providing strong evidence for the universality of these principles across diverse glassy materials. We further show that these principles manifest as a wavenumber-independent broad band in the dynamical structure factor, and we demonstrate that this band agrees well with inelastic X-ray scattering data for silica glass. These results advance understanding of the BP in network-forming glasses and offer practical criteria for interpreting scattering data.

## Results and Discussion

We perform classical molecular dynamics (MD) simulations of silica (${\text{SiO}}_{2}$) glass. We consider a three-dimensional system composed of $N_{\text{Si}}$ silicon (Si) atoms and $N_{\text{O}}$ oxygen (O) atoms, where $N_{\text{O}}=2N_{\text{Si}}$ and the total number of atoms is $N=N_{\text{O}}+N_{\text{Si}}=3N_{\text{Si}}$. The Si and O masses are $m_{\text{Si}}$ and $m_{\text{O}}$, respectively, with the ratio $m_{\text{Si}}/m_{\text{O}}=1.755$. Interatomic interactions are modeled with the SHIK potential (77), an extension of the van Beest–Kramer–van Santen (BKS) potential (78). BKS and SHIK are pairwise-additive potentials composed of a short-range two-body term $\phi_{S}$ and a long-range Coulomb term $\phi_{L}$. The SHIK potential has been carefully modified and extensively validated against experiments and first-principles simulations, reproducing thermodynamic quantities, structural observables (radial distribution function, static structure factor, bond-angle distribution), and elastic moduli (77, 79–82). These benchmarks establish SHIK as a reliable empirical potential for simulations of silica glass.

We fixed the mass density of silica glass at ρ=2.20 g/cm^3^ and controlled the temperature with a Nosé–Hoover thermostat (83, 84). Initial Si and O positions were randomized, and the system was equilibrated at T=3500 K for 100 ps to obtain a homogeneous liquid. The system was then cooled to T=300 K at a rate of 1 K/ps and equilibrated at T=300 K for 100 ps. Finally, all atomic velocities were set to zero and the configuration was relaxed by energy minimization to obtain the inherent structure $\vec{r}=\left[{\vec{r}}_{1},{\vec{r}}_{2},\ldots ,{\vec{r}}_{N}\right]$.

**Fig. 1.**
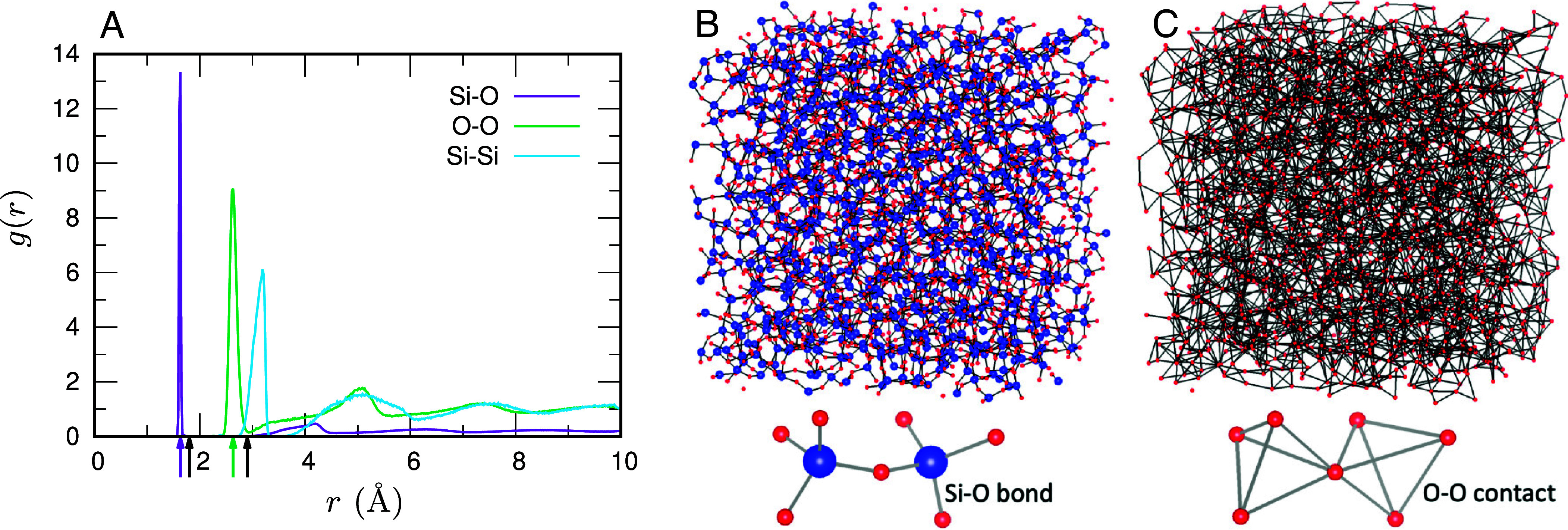
Atomic configuration in the inherent structure of silica glass. (*A*) The radial distribution function g(r) is plotted as a function of distance *r* for Si–O (purple), O–O (green), and Si–Si (cyan) pairs. For clarity, the Si–O curve is scaled by a factor of 0.2 (i.e., 0.2g(r)). The purple and green arrows mark the first peaks at r≈1.6 Å (Si–O) and r≈2.6 Å (O–O), which indicate the typical nearest-neighbor separations. The black arrows indicate the distance cutoffs used to define connectivity: r≤1.8 Å for Si–O bonds and r≤2.9 Å for O–O contacts, chosen at the first minima following these peaks. (*B*) Atomic configuration rendered with Si–O bonds. (*C*) Atomic configuration rendered with O–O contacts; only O atoms are shown to highlight the O network that outlines the tetrahedral units (Si centers not shown). The overall structure consists of corner-sharing ${\text{SiO}}_{4}$ tetrahedra.

Fig. 1*A* shows the radial distribution function g(r) of the inherent structure. The first peaks mark the typical nearest-neighbor separations for Si–O and O–O, at r≈1.6 Å and r≈2.6 Å, respectively (purple and green arrows). Throughout, we refer to Si–O nearest-neighbor pairs as Si–O bonds because they represent covalent Si–O linkages, whereas we refer to O–O nearest-neighbor pairs as O–O contacts because there is no covalent O–O bond; these contacts arise from the geometry of O–Si–O linkages, with the directionality of Si–O covalency represented implicitly by the pairwise potential (77, 78). Specifically, we define Si–O bonds as pairs with r≤1.8 Å and O–O contacts as pairs with r≤2.9 Å (black arrows). Panels (*B* and *C*) of Fig. 1 visualize the atomic network using these definitions. These visualizations confirm a tetrahedral network in which each Si atom sits at the center and four O atoms occupy the vertices, yielding four Si–O bonds and six O–O contacts per tetrahedral unit.

In addition to silica glass, we analyze HS, LJ, and soft-sphere (SS) glasses in three dimensions. The HS model, extensively studied in the context of the jamming transition (52–54), is a packing glass known to exhibit isostaticity and marginal stability together with the BP and QLVs (55–57, 60–65). We consider two HS preparations: a high-connectivity sample (HSH) generated at high pressure and a low-connectivity sample (HSL) generated at low pressure. Furthermore, LJ and SS glasses, representative van der Waals systems, have been investigated for their BP and QLVs in prior works (22, 23, 30, 31, 66–68).

In what follows, we present results for silica glass, HSH glass, and LJ glass in the main text. Complementary data for HSL and SS glasses are provided in *SI Appendix* to corroborate and strengthen our conclusions.

### Isostaticity.

**Fig. 2.**
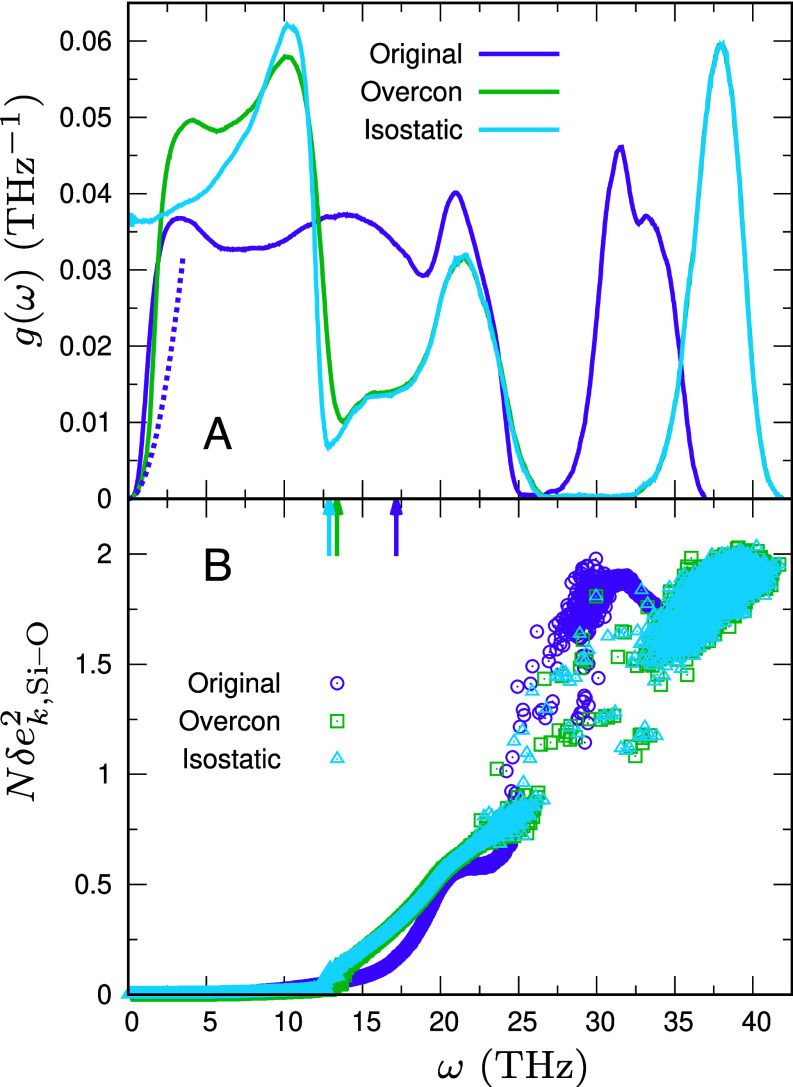
Vibrational states in silica glass. (*A*) g(ω) and (*B*) $N\delta e_{k,\text{Si–O}}^{2}$ are plotted as functions of frequency *ω* for the original system (purple), the overconstrained-network system (green), and the isostatic-network system (cyan). Vertical arrows mark the frequencies above which the cumulative number of modes reaches $4N_{\text{Si}}$ out of the total $9N_{\text{Si}}=3N$ modes. In (*A*), the purple dotted line indicates the Debye vDOS, $A_{D}\omega^{2}$, for the original system.

**Fig. 3.**
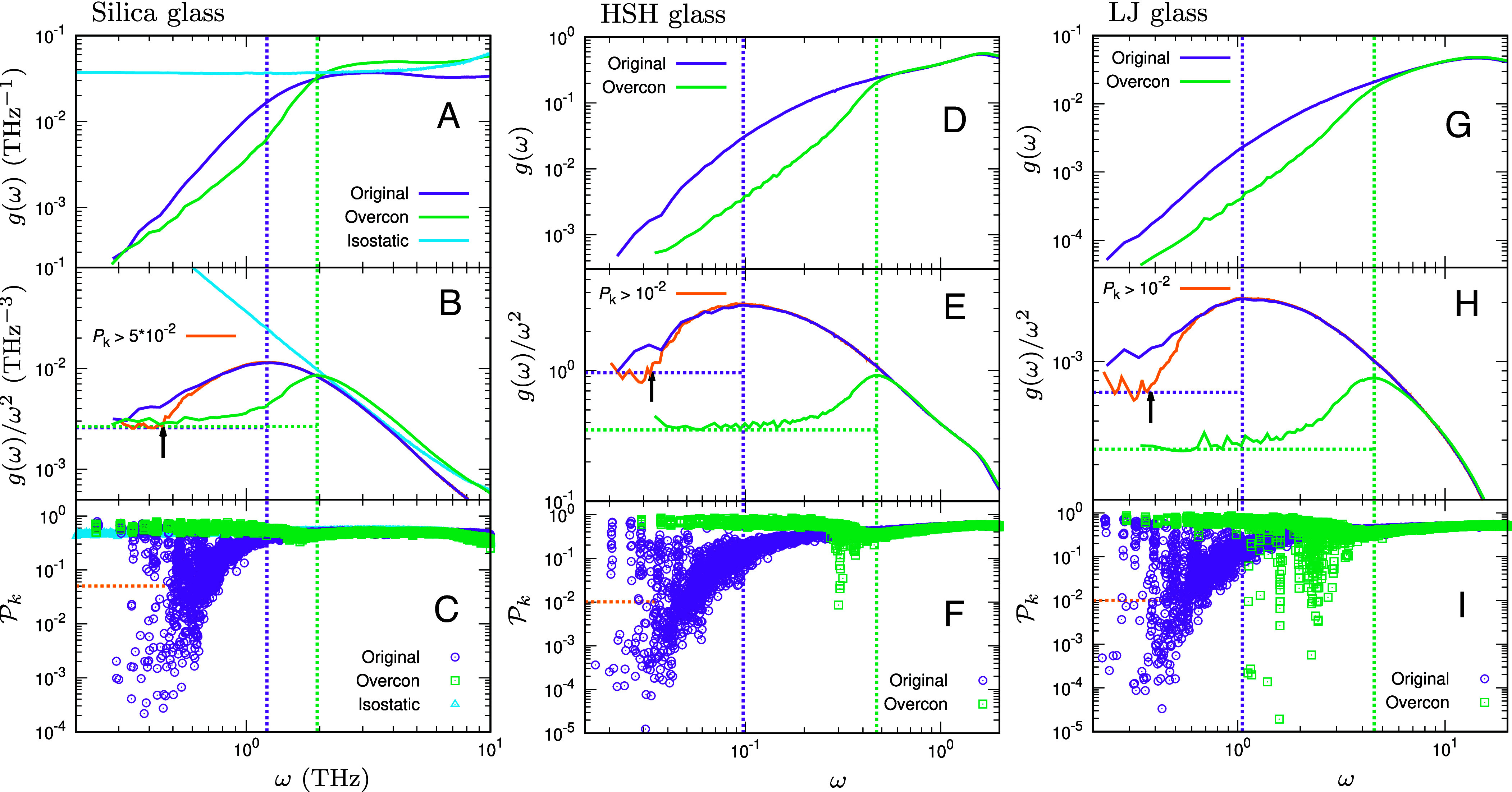
Vibrational states in the low-frequency regime. (*A*–*C*) Silica glass, (*D*–*F*) HSH glass, and (*G*–*I*) LJ glass. g(ω), $g\left(\omega \right)/\omega^{2}$, and $P_{k}$ are plotted as functions of *ω* for the original system (purple) and the overconstrained-network system (green). For silica glass (*A*–*C*), the isostatic-network system (cyan) is also shown. Vertical lines indicate the BP frequency $\omega_{\text{BP}}$ for the original and overconstrained-network systems, and horizontal dotted lines in (*B*, *E*, and *H*) indicate the Debye level $A_{D}$. Note that, for the isostatic-network system of silica glass, $\omega_{\text{BP}}\to 0$ and $A_{D}\to \infty$. Panels (*B*, *E*, and *H*) also show, in orange, the vDOS $g_{\text{EXT}}\left(\omega \right)$ of extended modes, defined by the participation-ratio threshold $P_{k}>P_{\text{th}}$ with $P_{\text{th}}=5\times 10^{-2}$ for silica glass and $P_{\text{th}}=10^{-2}$ for HSH and LJ glasses; these thresholds are marked by the horizontal dotted lines in (*C*, *F*, and *I*). Arrows in (*B*, *E*, and *H*) mark the continuum-limit frequency $\omega_{0}$ at which $g_{\text{EXT}}\left(\omega \right)/\omega^{2}$ converges to $A_{D}$. The vDOS $g_{\text{QLV}}\left(\omega \right)$ of QLVs, modes with $P_{k}\leq P_{\text{th}}$, is shown in Fig. 4. See also *SI Appendix*, Fig. S1 for HSL and SS glasses.

Fig. 2*A* displays the vDOS g(ω) as a function of frequency ω^*^ for silica glass (purple curve), which agrees with previously reported results (77, 81, 82, 85, 86). For reference, the Debye vDOS $A_{D}\omega^{2}$ is also shown (purple dotted curve); in the low-frequency regime g(ω) clearly exceeds this Debye vDOS, revealing excess modes. We further resolve the low-frequency behavior in Fig. 3 *A* and *B*. In Fig. 3*B*, the reduced vDOS $g\left(\omega \right)/\omega^{2}$ lies above the Debye level $A_{D}$, thereby confirming the presence of the BP. We define the BP frequency $\omega_{\text{BP}}$ as the frequency at which $g\left(\omega \right)/\omega^{2}$ attains its maximum. For silica glass, this yields $\omega_{\text{BP}}\approx 1.21$ THz, consistent with scattering experiments (8, 10, 13, 17).

To understand the vDOS and the BP, we begin by examining isostaticity in silica glass. As shown in Fig. 1, the Si and O atoms form a tetrahedral network with typical nearest-neighbor separations of 1.6 Å for Si–O bonds and 2.6 Å for O–O contacts. Accordingly, we construct a network system by connecting pairs with unstressed (neither prestretched nor precompressed) springs: Si–O pairs within the cutoff 1.8 Å are linked, and O–O pairs within 2.9 Å are likewise connected. Table 1 lists typical values of the spring constants $\phi_{S}^{\prime \prime }$ and $\phi_{L}^{\prime \prime }$, i.e., the second derivatives of the short-range and Coulomb potentials, respectively, evaluated at r=1.6 Å for Si–O and r=2.6 Å for O–O, together with the counts of pairs corresponding to Si–O bonds and O–O contacts. We first focus on the short-range spring $\phi_{S}^{\prime \prime }$, neglecting the Coulomb spring $\phi_{L}^{\prime \prime }$; that is, we analyze a network system in which these pairs are connected by springs with stiffness $\phi_{S}^{\prime \prime }$.

**Table 1.** Characteristics of Si–O bonds and O–O contacts in silica glass

Pair	Separation (Å)	Spring constant (eV Å^−2^)	Number of pairs
Si–O	1.6	$\phi_{S}^{\prime \prime }=35.8$	$4.00\;N_{\text{Si}}$
		$\phi_{L}^{\prime \prime }=-11.1$	
O–O	2.6	$\phi_{S}^{\prime \prime }=4.55$	$3.00\;N_{\text{O}}$
		$\phi_{L}^{\prime \prime }=1.29$	

“Separation” lists the typical nearest-neighbor separations. “Spring constant” lists $\phi_{S}^{\prime \prime }$ and $\phi_{L}^{\prime \prime }$, the second derivatives of the short-range and long-range Coulomb pair potentials, respectively. Neighbor (pair) definitions use distance cutoffs taken at r≤1.8 Å for Si–O bonds and r≤2.9 Å for O–O contacts. See also Fig. 1.

We can demonstrate that the network system constructed above is isostatic, meaning that the number of constraints $N_{\text{const}}$ equals the number of degrees of freedom $N_{\text{dof}}$. First, the total number of degrees of freedom is $N_{\text{dof}}=3\left(N_{\text{Si}}+N_{\text{O}}\right)=9N_{\text{Si}}$. Next, following ref. 44, we count constraints as follows. To match the situation in ref. 44 within our spring representation, we regard the O–O springs along tetrahedral edges as effective angular constraints associated with the ∠ O–Si–O bending (77, 78), and we treat only the Si–O springs as central-force bonds (this situation is visualized in Fig. 1*B*). In this representation, oxygen atoms are subject only to bond-length constraints, whereas silicon atoms experience both bond-length constraints and bond-angle constraints associated with ∠ O–Si–O. The total number of constraints is thus counted as (44)

[1] $$\begin{array}{c} \begin{array}{cc} N_{\text{const}} & =N_{\text{O}}\frac{z_{\text{O}}}{2}+N_{\text{Si}}\left[\frac{z_{\text{Si}}}{2}+\left(2z_{\text{Si}}-3\right)\right],\\ & =N_{\text{O}}\cdot 1+N_{\text{Si}}\cdot \left(2+5\right)=9N_{\text{Si}}=N_{\text{dof}}, \end{array} \end{array}$$

where $z_{\text{O}}=2$ and $z_{\text{Si}}=4$ are the coordination numbers of oxygen and silicon atoms, respectively. Eq. **1** confirms that the network representation is isostatic.

We present the vDOS for this isostatic-network system in Figs. 2*A* and 3*A* (cyan curves). The vDOS remains finite as ω→0, indicating a gapless spectrum at isostaticity that comprises both strictly zero-frequency floppy modes and many additional soft, low-frequency modes of isostatic origin (55–57).

A notable point is that Si–O bonds are significantly stiffer than O–O contacts: $\phi_{S}^{\prime \prime }=35.8$ eV Å^−2^ for Si–O versus $\phi_{S}^{\prime \prime }=4.55$ eV Å^−2^ for O–O, as listed in Table 1. Consequently, vibrations that involve stretching and compression of Si–O bonds appear at high frequencies (85). Since each tetrahedral unit contains four Si–O bonds, the total number of such modes can reach $4N_{\text{Si}}$. To probe these modes, we quantify Si–O bond stretching and compression using the dimensionless measure $\delta e_{k,\text{Si–O}}^{2}$ (*Materials and Methods*). Fig. 2*B* plots $N\delta e_{k,\text{Si–O}}^{2}$ versus *ω*. $N\delta e_{k,\text{Si–O}}^{2}$ remains nearly zero in the low-*ω* regime, including the BP region, indicating minimal Si–O bond deformation there. Above ω≈12.5 THz, however, $N\delta e_{k,\text{Si–O}}^{2}$ increases, signaling the onset of bond-stretching/compression character. In addition, the number of modes above ω≈12.5 THz is approximately $4N_{\text{Si}}$. Thus ω≈12.5 THz marks a boundary: Modes above this frequency predominantly occupy the $4N_{\text{Si}}$ sector associated with Si–O bond stretching and compression, whereas modes below it constitute the remaining $5N_{\text{Si}}$ modes.

Because the short-range interaction $\phi_{S}^{\prime \prime }$ couples not only atoms within a tetrahedral unit but also atoms in different units, it is natural to consider spring-network variants that deviate from isostaticity. These interunit couplings correspond to van der Waals interactions and are often referred to as weak interactions or bonds (43, 44, 48, 55, 66). Specifically, we examine a network in which all O–O pairs within the potential cutoff distance $r_{\mathrm{cS}}=8.0$ Å of $\phi_{S}$ are connected by unstressed springs with stiffness $\phi_{S}^{\prime \prime }$. In this case, the system is overconstrained, $N_{\text{const}}>N_{\text{dof}}$, and the vDOS vanishes as ω→0. We refer to this overconstrained variant as the overconstrained-network system. As shown in Fig. 2, the excess constraints shift the soft, low-frequency modes in the isostatic-network system to higher frequencies in the overconstrained-network system (compare the cyan and green curves). By contrast, the high-frequency modes associated with stretching and compression of Si–O bonds above ω≈12.5 THz are minimally affected, and the $N\delta e_{k,\text{Si–O}}^{2}$ data show little difference between the two network systems.

Finally, to transition from the overconstrained-network system back to the original atomistic system, we must reintroduce effects neglected so far: the short-range Si–Si springs $\phi_{S}^{\prime \prime }$, the long-range Coulomb springs $\phi_{L}^{\prime \prime }$, and the internal stresses introduced when unstressed springs are replaced by stressed (stretched or compressed) springs (55–57, 61, 63, 66, 71, 72). Note that the internal stresses originate from the first derivatives of the potentials, $\phi_{S}^{\prime }$ and $\phi_{L}^{\prime }$. While their consequences for low-frequency modes are discussed in the next section, here we focus on their impact at high frequencies. As seen in Fig. 2, the overconstrained-network system exhibits a clear separation: The band of modes dominated by stretching and compression of Si–O bonds (up to $4N_{\text{Si}}$ modes) is well separated from the remaining $5N_{\text{Si}}$ modes. In the original system, this separation is less pronounced. A key reason is that the Coulomb contribution, characterized by $\phi_{L}^{\prime \prime }$, reduces the effective stiffness of Si–O bonds: $\phi_{S}^{\prime \prime }=35.8$ eV Å^−2^ is lowered to $\phi_{S}^{\prime \prime }+\phi_{L}^{\prime \prime }=35.8-11.1=24.7$ eV Å^−2^ (Table 1). This weakening shifts the Si–O bond-stretching/compression band toward lower frequencies. Nevertheless, the trend persists in the original system: The $N\delta e_{k,\text{Si–O}}^{2}$ data show that bond stretching and compression continue to dominate the high-frequency region, whereas modes without such bond deformation dominate at low frequencies.

### Marginal Stability.

We now examine marginal stability in silica glass. Since the concept of marginal stability is well established in packing-based glasses (52–64, 66–72), we discuss it alongside data for HSH and LJ glasses. Fig. 3 presents g(ω), $g\left(\omega \right)/\omega^{2}$, and the participation ratio $P_{k}$ (*Materials and Methods*). The quantity $P_{k}$ measures the fraction of particles participating in the mode *k* (87). As limiting cases, $P_{k}=1$ corresponds to a fully extended mode in which all particles vibrate equally, whereas $P_{k}=1/N$ corresponds to a localized mode involving a single particle.

We present data for silica glass in panels (*A*–*C*) of Fig. 3. In the isostatic-network system (cyan), the vDOS approaches a nonzero constant as ω→0 (i.e., $g\left(\omega \right)\propto \omega^{0}$); accordingly, $g\left(\omega \right)/\omega^{2}\propto \omega^{-2}\to \infty$, and the BP collapses to zero frequency. In the isostatic state, the low-frequency sector comprises both strictly floppy modes at zero frequency and additional soft, nonzero-frequency modes that likewise originate from isostaticity (though they are not classified as floppy) (55–57). The participation-ratio data indicate that these low-frequency vibrational states are spatially extended.

Turning to the overconstrained-network system (green), the excess constraints cause the vDOS to vanish as ω→0. As seen in panel (*B*), $g\left(\omega \right)/\omega^{2}$ displays a clear BP. Below the BP frequency $\omega_{\text{BP}}$, $g\left(\omega \right)/\omega^{2}$ converges to the Debye level $A_{D}$, hence $g\left(\omega \right)\to A_{D}\omega^{2}$, and the vibrational states are extended phonons with large $P_{k}$. Above $\omega_{\text{BP}}$, the soft modes of isostatic origin (zero-frequency floppy modes and additional nonzero-frequency modes) are lifted to finite frequencies and merge into a nonphononic band. This band remains spatially extended with large $P_{k}$, and its accumulation produces the excess over the Debye law, i.e., the BP (55–57, 66).

**Fig. 4.**
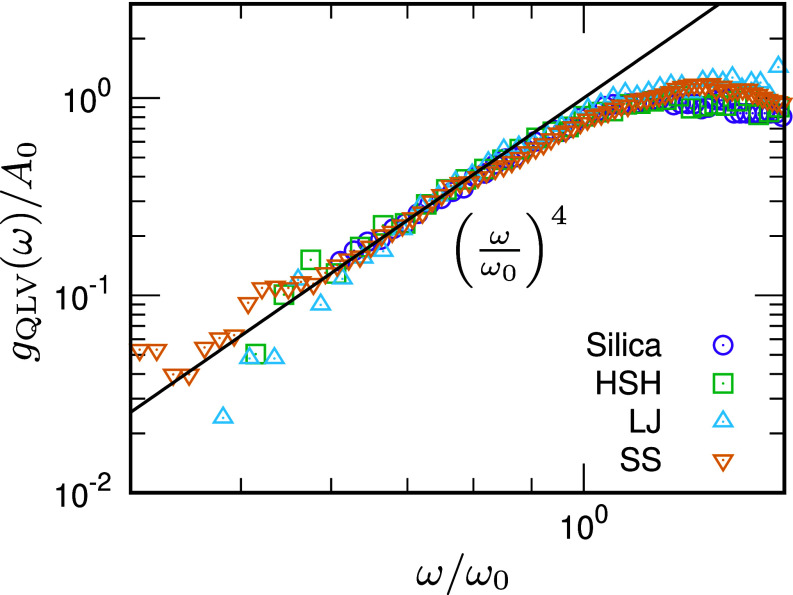
Vibrational density of states of quasi-localized vibrations (QLVs). $g_{\text{QLV}}\left(\omega \right)/A_{0}$ is plotted as a function of $\omega /\omega_{0}$ for silica glass (circles), HSH glass (squares), LJ glass (upward triangles), and SS glass (downward triangles). The solid line indicates $g_{\text{QLV}}\left(\omega \right)=A_{0}{\left(\omega /\omega_{0}\right)}^{4}\propto \omega^{4}$ for $\omega <\omega_{0}$. The prefactor is $A_{0}=4.62\times 10^{-4}$ THz^−2^, $1.38\times 10^{-3}$, $6.90\times 10^{-5}$, and $3.10\times 10^{-4}$ for silica, HSH, LJ, and SS glasses, respectively.

Finally, to move from the overconstrained-network system to the original atomistic system, we reinstate all effects neglected in constructing the overconstrained network, including the internal stresses introduced by replacing unstressed springs with stressed ones. With these ingredients restored, the isostaticity-derived band of soft modes shifts toward lower frequencies, and the BP accordingly moves downward as $\omega_{\text{BP}}$ decreases (purple). Notably, QLVs with low $P_{k}$ emerge at the low-*ω* edge below $\omega_{\text{BP}}$. Following previous works (63, 67), we partition modes into extended modes with $P_{k}>P_{\text{th}}$ and QLVs with $P_{k}\leq P_{\text{th}}$, and we compute their vDOSs, $g_{\text{EXT}}\left(\omega \right)$ and $g_{\text{QLV}}\left(\omega \right)$, separately. Here we take $P_{\text{th}}=5\times 10^{-2}$ for silica glass.^†^ We observe that $g_{\text{EXT}}\left(\omega \right)$ converges to the Debye law $A_{D}\omega^{2}$ at a characteristic frequency $\omega_{0}$ [orange curve in panel (*B*)]. Furthermore, $g_{\text{QLV}}\left(\omega \right)$ follows $A_{0}{\left(\omega /\omega_{0}\right)}^{4}\propto \omega^{4}$, as shown in Fig. 4. Previous simulations (74, 76) have also reported $g_{\text{QLV}}\propto \omega^{4}$ using different statistical analyses of QLVs.^‡^ Importantly, the QLVs exhibit a gapless vDOS with a power-law dependence on *ω*, indicating that silica glass is driven into a marginally stable state.

Fig. 3 additionally presents data for HSH glass in panels (*D*–*F*) and LJ glass in panels (*G*–*I*). For each system, we analyze an overconstrained-network counterpart in which interacting pairs (contacts for HSH and neighbors within the potential cutoff for LJ) are connected by unstressed springs with stiffness set by the pairwise force constants $\phi^{\prime \prime }$, thus removing the internal stress (prestress) and realizing the “unstressed” systems used in previous works (55–57, 61, 63, 66, 71, 72). Examining panels (*E* and *H*), both overconstrained-network systems (green) exhibit a clear BP: Below $\omega_{\text{BP}}$, g(ω) converges to the Debye law and the vibrational states are extended phonons with large $P_{k}$,^§^ whereas above $\omega_{\text{BP}}$ a nonphononic band of spatially extended soft modes with large $P_{k}$ produces the excess over the Debye law, i.e., the BP (55–57, 66). When internal stresses are introduced by replacing unstressed springs with stressed ones, this band of soft modes shifts to lower frequencies and the BP correspondingly moves downward as $\omega_{\text{BP}}$ decreases (purple), while QLVs with low $P_{k}$ emerge at the low-*ω* edge below $\omega_{\text{BP}}$. Following the procedure used above for silica glass (63, 67), we compute $g_{\text{EXT}}\left(\omega \right)$ and $g_{\text{QLV}}\left(\omega \right)$ by setting $P_{\text{th}}=10^{-2}$. As shown in panels (*E* and *H*), $g_{\text{EXT}}\left(\omega \right)$ follows the Debye law for $\omega <\omega_{0}$ (see orange curves). The same behavior is observed for SS glass in *SI Appendix*, Fig. S1. Furthermore, for all of HSH, LJ, and SS glasses, $g_{\text{QLV}}\left(\omega \right)$ follows the gapless form $A_{0}{\left(\omega /\omega_{0}\right)}^{4}\propto \omega^{4}$, as shown in Fig. 4, consistent with previous studies (63, 67, 68). Taken together, these results indicate that these systems are driven toward marginal stability.

Silica glass, HS glass, LJ glass, and SS glass all share a characteristic tendency toward marginal stability. In HS, LJ, and SS glasses, internal stresses arising from short-range interactions, namely contact forces or van der Waals forces, drive the systems toward marginal stability. By contrast, in silica glass the approach to marginal stability is driven collectively by the short-range Si–Si interaction, the long-range Coulomb interaction, and the internal stresses associated with the $\phi_{S}^{\prime }$ and $\phi_{L}^{\prime }$ terms. While all of these ingredients are required, the internal stresses appear to be the essential factor controlling the proximity to marginal stability. Moreover, among the internal stresses, those linked to short-range repulsive forces ($\phi_{S}^{\prime }$) tend to destabilize the system, whereas those associated with the attractive Coulomb interaction between Si and O atoms ($\phi_{L}^{\prime }$) act to stabilize it and partially offset the repulsive contribution. Consequently, in silica glass the difference in BP frequency between the original system and the overconstrained-network system is modest, about a factor of 1.6 (compare the green and purple vertical lines in Fig. 3*B*), whereas the corresponding shift is larger, by a factor of 4 to 8, in HS, LJ, and SS glasses.

### Dynamical Structure Factor.

Finally, we examine how isostaticity and marginal stability are reflected in the dynamical structure factor $S_{\alpha }\left(q,\omega \right)$, with α=T,L denoting the transverse and longitudinal polarizations, which is accessible via inelastic scattering experiments. In the low-frequency regime, the vDOS can be estimated by integrating $S_{\alpha }\left(q,\omega \right)$ over wavenumber *q* up to the Debye wavenumber $q_{D}$:

[2] $$\begin{array}{c} \frac{g(\omega )}{\omega^{2}}=\frac{2\;M(\omega )}{q_{D}^{3}}\int_{0}^{q_{D}}\left\{\frac{S_{T}\left(q,\omega \right)}{k_{B}T}+\frac{S_{L}\left(q,\omega \right)}{k_{B}T}\right\}\;dq, \end{array}$$

where $k_{B}$ is the Boltzmann constant and M(ω) is the effective mass (see *SI Appendix* for the derivation of Eq. **2**). Setting $\omega =\omega_{\text{BP}}$ allows us to resolve how excitations at each wavenumber *q* in $S_{T}\left(q,\omega_{\text{BP}}\right)$ and $S_{L}\left(q,\omega_{\text{BP}}\right)$ contribute to $g\left(\omega_{\text{BP}}\right)/\omega_{\text{BP}}^{2}$, i.e., the BP. This viewpoint underlies theoretical analyses (14, 29, 30, 32, 58, 59, 64), which employ Green’s functions related to $S_{\alpha }\left(q,\omega \right)$ via the fluctuation–dissipation theorem.

**Fig. 5.**
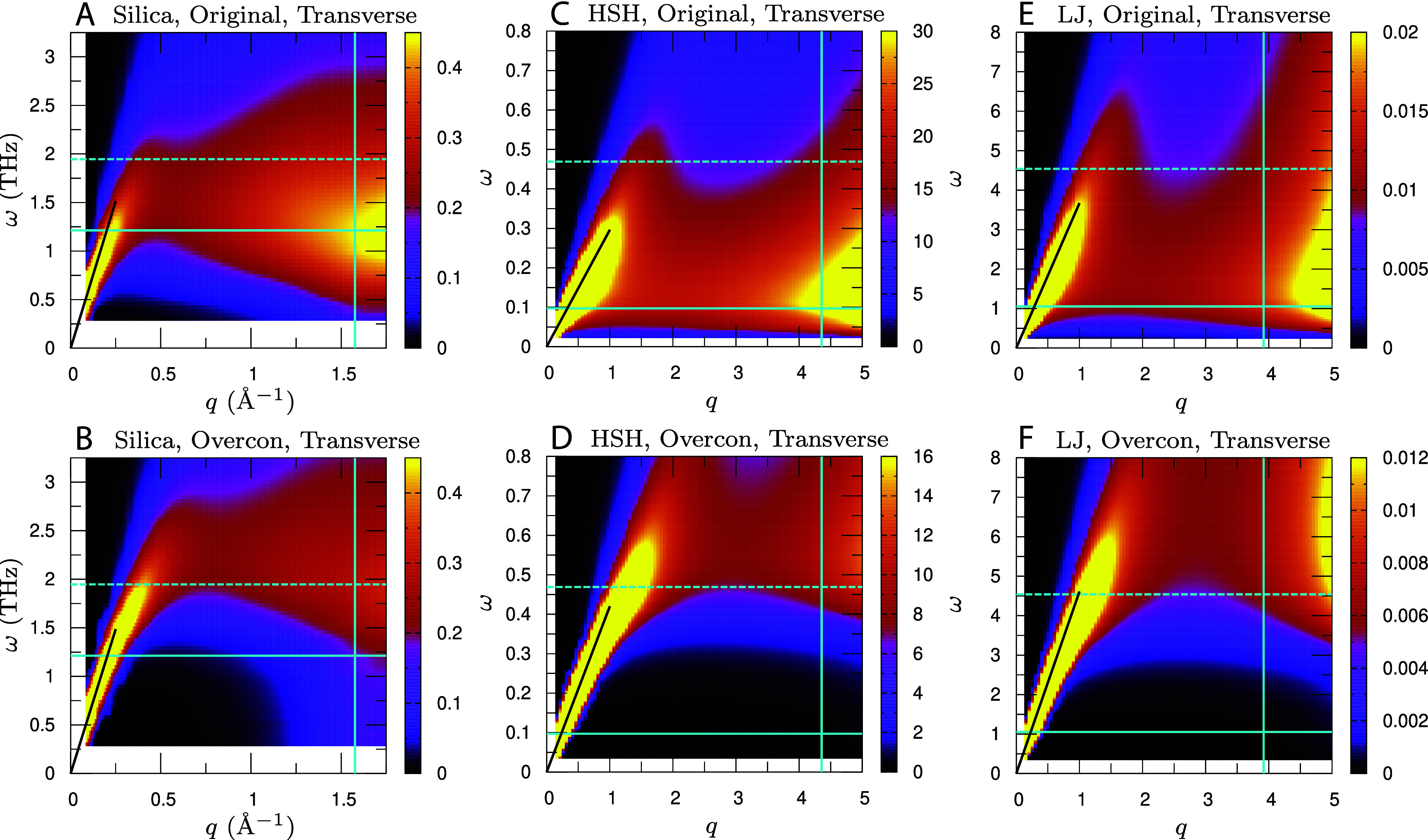
Transverse dynamical structure factor. (*A* and *B*) Silica glass, (*C* and *D*) HSH glass, and (*E* and *F*) LJ glass. $S_{T}\left(q,\omega \right)/\left(k_{B}T\right)$ is shown as a function of *q* and *ω* for the original systems in (*A*, *C*, and *E*) and for the overconstrained-network systems in (*B*, *D*, and *F*). For silica glass, values are reported in units of ${\left(\mathrm{eV}\mathrm{THz}\right)}^{-1}$. The vertical line marks the Debye wavenumber $q_{D}$. Horizontal solid and dashed lines indicate the BP frequency $\omega_{\text{BP}}$ for the original and overconstrained-network systems, respectively. The black solid curve shows the linear dispersion $\omega =c_{T}q$, with $c_{T}$ being the transverse sound speed, corresponding to phonon excitations. Data for the isostatic-network system of silica glass are provided in *SI Appendix*, Fig. S3. Additional results for HSL and SS glasses are provided in *SI Appendix*, Fig. S4.

Fig. 5 presents the transverse dynamical structure factor $S_{T}\left(q,\omega \right)$, focusing on the low-frequency and low-wavenumber ($q≲q_{D}$) regime. In the figure, data for the overconstrained-network system are shown alongside those for the original system. We first examine silica glass, shown in panels (*A* and *B*). For silica glass, data for the isostatic-network system are also provided in *SI Appendix*, Fig. S3, where isostaticity produces a nearly wavenumber-independent band that extends to the zero-frequency limit. The transverse $S_{T}\left(q,\omega \right)$ (also longitudinal $S_{L}\left(q,\omega \right)$) accumulate nearly *q*-independent spectral weight that diverges as ω→0. Consistently, $g\left(\omega \right)/\omega^{2}\to \infty$, as indicated by Eq. **2**.

Moving to the overconstrained-network system in panel (*B*) of Fig. 5, phonon excitations appear at low *ω* and low *q* along the linear dispersion $\omega =c_{T}q$, where $c_{T}$ is the transverse sound speed. In addition, a broad, approximately wavenumber-independent band emerges around and above the BP and extends up to $q_{D}$, showing nonphononic excitations. This band originates from isostaticity-derived modes that, in the presence of excess constraints, are lifted to finite frequencies (Fig. 3). Therefore, the BP is built from two components: linearly dispersing phonons that follow $\omega =c_{T}q$ and a nearly dispersionless band of isostaticity-derived modes.

Finally, turning to the original atomistic system in panel (*A*), restoring the internal stresses arising from short-range and long-range Coulomb forces shifts the entire nonphononic band to lower frequencies, and $\omega_{\text{BP}}$ decreases accordingly. At the low-*ω* edge below $\omega_{\text{BP}}$, a band of QLV excitations appears (63, 67, 68). Phonon ridges along the linear dispersion $\omega =c_{T}q$ remain visible, but they are broader than in the overconstrained-network system. This broadening is attributed to isostaticity-derived modes shifting into the low-frequency range and hybridizing with the phonons, which increases the phonon linewidth [an effect that is even more pronounced for HSH in panel (*C*) and for LJ glass in panel (*E*)]. In this way, marginal stability manifests as a wavenumber-independent band that is shifted downward by internal stresses, together with the emergence of QLV excitations at the low-frequency edge.

Fig. 5 also presents data for HSH glass in panels (*C* and *D*) and LJ glass in panels (*E* and *F*). For the overconstrained-network (unstressed) systems in panels (*D* and *F*), a phonon branch appears along $\omega =c_{T}q$, and a broad, approximately wavenumber-independent band emerges around and above the BP. Turning to the original systems that include internal stress (prestress) in panels (*C* and *E*), the entire nonphononic band shifts to lower frequencies, with a corresponding decrease in $\omega_{\text{BP}}$, and QLVs form a broad band at the low-*ω* edge below $\omega_{\text{BP}}$. These behaviors are also observed for HSL and SS glasses, as shown in *SI Appendix*, Fig. S4. Notably, a broad, dispersionless band in the dynamical structure factor has also been reported recently in simulations of LJ glasses (91), consistent with the present results. As discussed above, in silica glass the attractive Coulomb forces between Si and O stabilize the system and partially offset the destabilizing effect of short-range forces. Consequently, the downward shift of the band is smaller in silica glass than in HS (HSH and HSL), LJ, and SS glasses. Despite these quantitative differences, the underlying physics remains the same across all the studied systems.

**Fig. 6.**
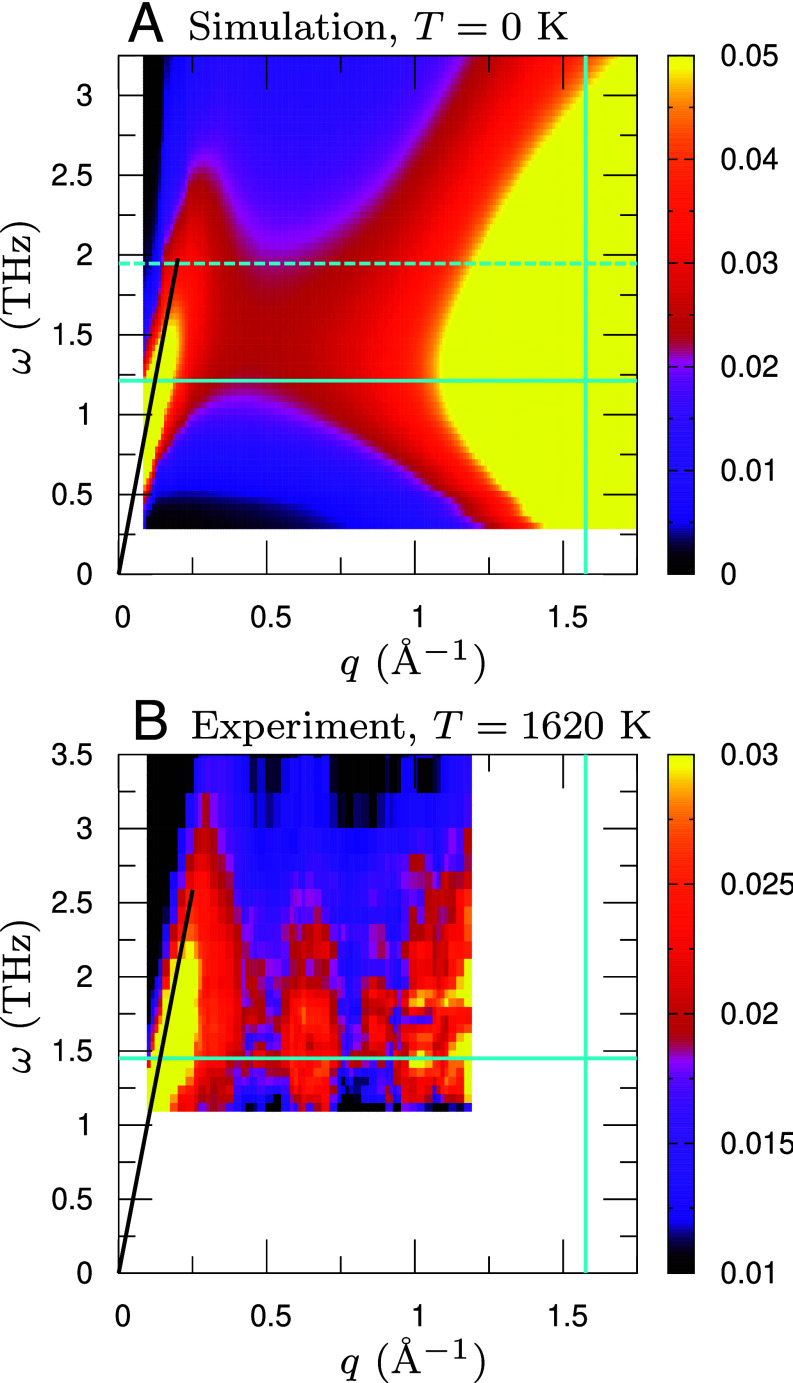
Longitudinal dynamical structure factor of silica glass. (*A*) Simulation result and (*B*) inelastic X-ray scattering data for the inelastic contribution. For the simulation in (*A*), $S_{L}\left(q,\omega \right)/\left(k_{B}T\right)$ is shown as a function of *q* and *ω* in units of ${\left(\mathrm{eV}\mathrm{THz}\right)}^{-1}$ for the original system. The density is ρ=2.20 g/cm^3^ and the temperature is T=0 K. The vertical line marks the Debye wavenumber $q_{D}=1.58$ Å^−1^. Horizontal solid and dashed lines indicate the BP frequency, $\omega_{\text{BP}}=1.21$ THz and 1.95 THz for the original and overconstrained-network systems, respectively. The black solid curve shows the linear dispersion $\omega =c_{L}q$, with $c_{L}$ being the longitudinal sound speed, corresponding to phonon excitations. Additional results for HS (HSH and HSL), LJ, and SS glasses are provided in *SI Appendix*, Fig. S5. For the experiment in (*B*), we plot $S_{L}\left(q,\omega \right)/\left[\hbar \omega (n(\omega ,T)+1)\right]$ (which reduces to $S_{L}\left(q,\omega \right)/\left(k_{B}T\right)$ in the classical limit) in units of ${\left(\mathrm{eV}\mathrm{THz}\right)}^{-1}$. The data correspond to ambient-pressure silica glass at density ρ=2.20 g/cm^3^ and temperature T=1,620 K. The vertical line again marks the Debye wavenumber $q_{D}=1.58$ Å^−1^, as in (*A*). The horizontal line marks the BP frequency $\omega_{\text{BP}}=1.45$ THz. The black curve shows the linear dispersion $\omega =c_{L}q$, using $c_{L}\approx 6,500$ m/s measured by Brillouin light scattering (13). Note that the experimental spectrum is limited to frequencies ω≳1 THz and wavenumbers q≲1.2 Å^−1^.

An intriguing aspect is the longitudinal channel, $S_{L}\left(q,\omega \right)$, which is shown for silica glass in Fig. 6*A* and for HS, LJ, and SS glasses in *SI Appendix*, Fig. S5. Across silica, HS, LJ, and SS glasses, $S_{L}\left(q,\omega \right)$ is markedly weaker than $S_{T}\left(q,\omega \right)$ in the low-frequency regime including the BP region (compare the color-scale ranges of $S_{T}$ and $S_{L}$ for each system), indicating that the BP predominantly reflects transverse motion (22, 23, 31, 64). In silica glass, however, $S_{L}\left(q,\omega \right)$ shows a similar *ω*–*q* structure to $S_{T}\left(q,\omega \right)$: A broad, nearly wavenumber-independent band appears around and above the BP as shown in Fig. 6*A*, whereas the corresponding feature is much less apparent in HS, LJ, and SS glasses (*SI Appendix*, Fig. S5). This behavior likely reflects closer proximity to isostaticity in silica glass, which enhances the population of isostaticity-derived soft modes with both transverse and longitudinal character. Moreover, silica glass exhibits strong nonaffine elasticity not only under shear but also under volumetric deformation (*Materials and Methods*), further reducing the contrast between transverse and longitudinal responses.

Since scattering experiments probe the longitudinal channel, we now compare our simulation data with the inelastic X-ray scattering data (11, 13, 14, 16, 18). Fig. 6*B* shows the measured longitudinal dynamical structure factor for ambient-pressure silica glass at density ρ=2.20 g/cm^3^ and temperature T=1620 K. The plotted quantity is the inelastic part of the experimentally determined dynamical structure factor, $S_{L}\left(q,\omega \right)/\left[\hbar \;\omega \;(n(\omega ,T)+1)\right]$ (which reduces to $S_{L}\left(q,\omega \right)/\left(k_{B}T\right)$ in the classical limit), in units of ${\left(\mathrm{eV}\mathrm{THz}\right)}^{-1}$, where n(ω,T) is the Bose–Einstein population factor and ħ=h/2π with *h* the Planck constant. Details on the procedure used to properly normalize the inelastic X-ray scattering data are provided in ref. 18. The data are limited to frequencies ω≳1 THz and wavenumbers q≲1.2 Å^−1^ because subtracting the elastic line becomes delicate at lower frequencies and at around the peak of the static structure factor. We also note that our simulation data in Fig. 6*A* correspond to T=0 K; accordingly, the BP frequency is $\omega_{\text{BP}}\approx 1.21$ THz in simulation but $\omega_{\text{BP}}\approx 1.45$ THz in the experiment, consistent with the expected upward shift at elevated temperature T=1,620 K. Despite this offset, the qualitative behavior is fully consistent between simulation and experiment: (i) at low wavenumber and frequency, longitudinal phonons follow the linear dispersion $\omega =c_{L}q$; and (ii) a broad, wavenumber-independent, nonphononic band is visible around the BP, extending from q≈0.3Å^−1^ up to ≈1.2 Å^−1^. By direct analogy with the simulation data, we identify this dispersionless band in the inelastic X-ray scattering data as the band of isostaticity-derived modes.

## Conclusion

We have explained the BP in silica glass in terms of isostaticity and marginal stability. When the tetrahedral covalent network structure is extracted, it forms an isostatic-network system in which the number of constraints equals the number of degrees of freedom. This isostatic network comprises both zero-frequency floppy modes and many additional soft, low-frequency modes of isostatic origin, and the vDOS remains finite as ω→0, i.e., $g\left(\omega \right)\propto \omega^{0}$. In practice, interunit couplings arising from van der Waals interactions introduce additional constraints beyond the degrees of freedom. The resulting spring network is overconstrained and supports phonons whose vDOS follows the Debye law, i.e., $g\left(\omega \right)≃A_{D}\omega^{2}$, while the soft modes of isostatic origin are shifted to higher frequencies, producing the nonphononic excess that constitutes the BP. Reinstating all effects neglected in the overconstrained-network construction, including frustration-induced internal stresses, shifts the nonphononic band downward, producing a lower-frequency BP. Concurrently, QLVs appear at the low-frequency edge below the BP and exhibit a gapless power-law vDOS $\propto \omega^{4}$. These features indicate that internal stresses drive silica glass to a marginally stable state, as also observed in HS, LJ, and SS glasses.

Furthermore, we find that isostaticity and marginal stability are encoded in the dynamical structure factor as a broad, nearly wavenumber-independent band around the BP, arising from isostaticity-derived soft modes. In silica glass, this band is clearly visible in both the transverse and longitudinal dynamical structure factors, whereas in HS, LJ, and SS glasses it is prominent in the transverse channel but much less apparent in the longitudinal one. This contrast is especially useful because scattering experiments generally cannot access the transverse component and instead probe the longitudinal component. Indeed, a direct comparison between inelastic X-ray scattering measurements and our simulations of silica glass shows good agreement in the longitudinal dynamical structure factor, including both the low-frequency phonon branch and the broad dispersionless band around the BP. On this basis, we identify the dispersionless band observed in the inelastic X-ray scattering data as the band of isostaticity-derived modes. Thus, the BP can be analyzed directly via the longitudinal dynamical structure factor, informing interpretations of existing measurements (4–18) and guiding future investigations. Notably, a recent paper (92) revisits existing experimental and simulation data from the perspective of dispersionless bands (excitations).

In conclusion, isostaticity and marginal stability are broadly applicable to both packing-based and network-forming glasses and capture fundamental aspects of the physics of amorphous solids. These principles manifest as a dispersionless, nearly wavenumber-independent band in the dynamical structure factor, providing a direct, testable signature. We also carried out an effective-medium mean-field analysis based on random spring networks, following refs. 58 and 59, and computed the vDOS and the dynamical structure factor; the results and explanations are reported in *SI Appendix*, Figs. S6 and S7. For the overconstrained-network system (often termed the “unstressed system”), the analysis predicts an accumulation of isostaticity-derived soft modes at finite frequencies that produces the BP. Crucially, when internal stress (or prestress) is included, these soft modes shift to lower frequencies and imprint themselves on the dynamical structure factor as a broad, wavenumber-independent band around the BP. These effective-medium predictions are in excellent agreement with our simulations and with the inelastic X-ray scattering data on silica glass, establishing a consistent picture across theory, simulation, and experiment.

Finally, we remark on a few important points. We have employed the SHIK potential, in which the O–Si–O and Si–O–Si angular constraints are effectively incorporated through suitably parameterized two-body interactions (77). Nevertheless, it is important to also study silica glass using a potential that explicitly includes angular terms, such as the Vashishta potential (93), and to confirm that our results are insensitive to whether covalent directionality is treated explicitly or implicitly. In *SI Appendix*, Figs. S8–S15, we analyze silica glass modeled with the Vashishta potential and find that the main results and conclusions obtained for the SHIK system are also reproduced with the Vashishta potential. This indicates that our results are robust, do not depend on the specific potential model, and, in particular, that explicitly including three-body interactions does not alter our results concerning isostaticity and marginal stability in silica glass.

From a methodological point of view, we introduce a logically controlled analysis of interatomic interactions to reveal isostaticity and marginal stability. This analysis is, in principle, applicable to any covalent network glass and thus opens a way to systematically investigate isostaticity and marginal stability in a broad class of covalent network systems. Indeed, the present method is straightforwardly applicable also to silica glass modeled with the Vashishta potential (93) and enables us to reveal isostaticity and marginal stability irrespective of the specific potential model. We therefore regard this method as a generally applicable tool for studying covalent network glasses.

By analogy with jammed packings, we expect that a length scale associated with isostaticity should also exist in silica glass. Such a length scale could be identified, for example, in the response to applied dipolar forces (94) or shear deformation (95), where it would appear as the crossover length beyond which continuum elasticity is recovered. In addition, in jammed packings it is known that spatial fluctuations of the contact number are strongly suppressed and the system is hyperuniform (96). By analogy, it is natural to expect that, in silica glass, spatial fluctuations of the Si–O covalent bonds are also suppressed, possibly leading to a hyperuniform structure. Exploring these possibilities would be a very interesting direction for future work.

## Materials and Methods

### Silica Glass.

Silica glass consists of $N_{\text{Si}}$ silicon (Si) atoms and $N_{\text{O}}$ oxygen (O) atoms in three-dimensional space, where $N_{\text{O}}=2N_{\text{Si}}$ and the total number of atoms is $N=N_{\text{O}}+N_{\text{Si}}=3N_{\text{Si}}$. The masses of the Si atom and the O atom are denoted as $m_{\text{Si}}$ and $m_{\text{O}}$, respectively, with the ratio $m_{\text{Si}}/m_{\text{O}}=1.755$. The interatomic forces are modeled using the BKS family of potentials, originally proposed in ref. 78 and subsequently modified and extended in several aspects (77, 79–82). BKS-type models are pairwise-additive and comprise a short-range two-body term and a long-range Coulomb term, and therefore they do not include explicit many-body angular contributions that encode covalent directionality. For a more explicit treatment of covalent bonding, many-body potentials with angular terms have been developed, notably the Vashishta potential (93), which adds three-body interactions associated with O–Si–O and Si–O–Si angles. In this study we adopt the SHIK parameterization of the BKS family (77), which has been rigorously tested to reproduce experimental data and first-principles simulations, including thermodynamic quantities, structural properties (radial distribution function, static structure factor, bond-angle distribution), and elastic moduli (77, 79–82). We also analyze silica glass modeled with the Vashishta potential; the results are presented in *SI Appendix*, Figs. S8–S15 and lead to the same results and conclusions as those obtained with the SHIK potential.

The potential comprises two contributions: the short-range term and the long-range Coulomb term. The short-range term is defined as

[3] $$\begin{array}{c} v_{S}\left(r\right)=A_{\alpha \beta }\exp\left(-B_{\alpha \beta }r\right)-\frac{C_{\alpha \beta }}{r^{6}}+\frac{D_{\alpha \beta }}{r^{24}}, \end{array}$$

where *r* is the interparticle distance, and *α* and *β* denote either Si or O. The parameters $A_{\alpha \beta }$, $B_{\alpha \beta }$, $C_{\alpha \beta }$, and $D_{\alpha \beta }$ are specified in Table 2. The long-range Coulomb term is given by

[4] $$\begin{array}{c} v_{L}\left(r\right)=\frac{q_{\alpha }q_{\beta }}{r}, \end{array}$$

**Table 2.** Parameters for the potential of silica glass

*α*–*β*	$A_{\alpha \beta }$ (eV)	$B_{\alpha \beta }$ (Å^−1^)	$C_{\alpha \beta }$ (eV Å^6^)	$D_{\alpha \beta }$ (eV Å^24^)
Si–O	23,107.8	5.098	139.7	66.0
O–O	1,120.5	2.893	26.1	16,800.0
Si–Si	2,797.9	4.407	0.0	3,423,204.0

$q_{\text{Si}}=1.7755\;e$, $q_{\text{O}}=-q_{\text{Si}}/2$, $r_{\mathrm{cS}}=8.0$ Å, $r_{\mathrm{cL}}=10.0$ Å, and $\gamma_{S}=\gamma_{L}=0.2$ Å.

where the charge of silicon is set as $q_{\text{Si}}=1.7755\;e$ (with *e* the elementary charge) and, to ensure charge neutrality, the charge of oxygen is set as $q_{\text{O}}=-q_{\text{Si}}/2$. The potentials $v_{S}\left(r\right)$ and $v_{L}\left(r\right)$ are truncated at $r=r_{\mathrm{cS}}=8.0$ Å and $r=r_{\mathrm{cL}}=10.0$ Å, respectively. For $v_{L}\left(r\right)$, this truncation is referred to as Wolf truncation (79, 80). To prevent discontinuities in the potential and its force (the first derivative of the potential) at the cutoff distances, $v_{S}\left(r\right)$ and $v_{L}\left(r\right)$ are smoothed as follows:

[5] $$\begin{array}{c} \begin{array}{cc} \phi_{S}\left(r\right) & =\left[v_{S}\left(r\right)-v_{S}\left(r_{\mathrm{cS}}\right)-\left(r-r_{\mathrm{cS}}\right)v_{S}^{\prime }\left(r_{\mathrm{cS}}\right)\right]G_{\mathrm{cS}}\left(r\right),\\ \phi_{L}\left(r\right) & =\left[v_{L}\left(r\right)-v_{L}\left(r_{\mathrm{cL}}\right)\right]G_{\mathrm{cL}}\left(r\right), \end{array} \end{array}$$

with the smoothing functions

[6] $$\begin{array}{c} \begin{array}{cc} G_{\mathrm{cS}}\left(r\right) & =\;\exp\left[-\frac{\gamma_{S}^{2}}{{\left(r-r_{\mathrm{cS}}\right)}^{2}}\right],\\ G_{\mathrm{cL}}\left(r\right) & =\;\exp\left[-\frac{\gamma_{L}^{2}}{{\left(r-r_{\mathrm{cL}}\right)}^{2}}\right], \end{array} \end{array}$$

where $\gamma_{S}=\gamma_{L}=0.2$ Å.

We fixed the mass density to be ρ=2.20 g/cm^3^, and to cover a broad range of wavenumbers *q* and frequencies *ω*, we used several system sizes ranging from $N=1.5\times 10^{4}$ to $2.4\times 10^{5}$ atoms. For each system size, we performed three independent MD simulations to obtain three inherent-structure configurations, $\vec{r}=\left[{\vec{r}}_{1},{\vec{r}}_{2},\ldots ,{\vec{r}}_{N}\right]$, i.e., T=0 K configurations. All reported quantities are averaged over these three independent configurations.

### HS Glass.

HS glass is a one-component system in three dimensions that has been extensively studied in the context of the jamming transition (52–54, 60–65). Two particles *i* and *j* interact via the harmonic potential,

[7] $$\begin{array}{c} \phi \left(r\right)=\frac{\epsilon }{2}{\left(1-\frac{r}{\sigma }\right)}^{2}H\left(\sigma -r\right), \end{array}$$

where $r=r_{\mathrm{ij}}$ is the interparticle distance, *σ* is the particle diameter, and *H* is the Heaviside step function. All particles have the same mass *m*. Physical quantities are measured in units of length *σ*, mass *m*, and energy *ϵ*; accordingly, *q* and *ω* are reported in units of $\sigma^{-1}$ and $\sqrt{\epsilon /(m\sigma^{2})}$, respectively. We prepared HS glasses at pressures P=0.05 and 0.005, referred to as HSH and HSL in this paper. At the higher pressure P=0.05 (HSH), particles are more densely packed and exhibit higher connectivity, whereas at the lower pressure P=0.005 (HSL), particles are less dense and have lower connectivity. As with silica glass, we simulated multiple system sizes, ranging from $N=1.6\times 10^{4}$ to $1.024\times 10^{6}$ particles, to access broad ranges of *q* and *ω*.

### LJ Glass.

LJ glass is a one-component system in three dimensions, studied in previous works (22, 67, 97). Two particles *i* and *j* interact via the Lennard–Jones (LJ) potential,

[8] $$\begin{array}{c} v\left(r\right)=4\epsilon \left[{\left(\frac{\sigma }{r}\right)}^{12}-{\left(\frac{\sigma }{r}\right)}^{6}\right], \end{array}$$

where $r=r_{\mathrm{ij}}$ is the interparticle distance and *σ* is the particle diameter. The potential is truncated at $r=r_{c}=2.5\sigma$. To avoid artificial anharmonicities caused by the discontinuity at $r=r_{c}$ (98), we employ the smoothed form;

[9] $$\begin{array}{c} \phi \left(r\right)=v\left(r\right)-v\left(r_{c}\right)-\left(r-r_{c}\right)v^{\prime }\left(r_{c}\right), \end{array}$$

which ensures that both the potential and the force (the derivative of the potential) vanish smoothly at $r=r_{c}$. All particles have the same mass *m*. Physical quantities are measured in units of length *σ*, mass *m*, and energy *ϵ*. The number density is set to $\hat{\rho }=N/V=1.015$, where *V* is the system volume. We simulated several system sizes, ranging from $N=1.6\times 10^{4}$ to $1.024\times 10^{6}$.

### SS Glass.

SS glass is a binary mixture of large (*L*) and small (*S*) particles in three dimensions, which we have previously studied in refs. 23 and 31. Particles *i* and *j* of types *α* and *β* (α,β∈{L,S}) interact via a 12-inverse-power-law potential,

[10] $$\begin{array}{c} v\left(r\right)=\epsilon {\left(\frac{\sigma_{\alpha \beta }}{r}\right)}^{12}, \end{array}$$

where $r=r_{\mathrm{ij}}$, $\sigma_{\alpha \beta }=\left(\sigma_{\alpha }+\sigma_{\beta }\right)/2$, and $\sigma_{\alpha }$ is the diameter of the large or small species ($\sigma_{L}$ or $\sigma_{S}$). The size ratio is $\sigma_{S}/\sigma_{L}=0.7$, and the composition is equimolar with $x_{L,S}=N_{L,S}/N=1/2$ and $N=N_{L}+N_{S}$. As in the case of LJ glass, we employ the smoothed form ϕ(r) defined in Eq. **9**. All particles have the same mass *m*. Physical quantities are measured in units of length $\sigma ={\left(\sum_{\alpha ,\beta =L,S}x_{\alpha }x_{\beta }\sigma_{\alpha \beta }^{3}\right)}^{1/3}$, mass *m*, and energy *ϵ*. The number density is set to $\hat{\rho }=N/V=1.015$. We simulated several system sizes, ranging from $N=1.6\times 10^{4}$ to $1.024\times 10^{6}$.

### Vibrational Modes.

Using the inherent structure $\vec{r}=\left[{\vec{r}}_{1},{\vec{r}}_{2},\cdots ,{\vec{r}}_{N}\right]$, we perform a standard normal-mode analysis by solving the eigenvalue problem of the dynamical matrix (a 3N×3N matrix) to obtain the eigenvalues $\lambda_{k}$ and eigenvectors ${\vec{e}}_{k}=\left[{\vec{e}}_{k,1},{\vec{e}}_{k,2},\cdots ,{\vec{e}}_{k,N}\right]$ for modes k=1,2,⋯,3N (87). We remove the three zero-frequency translational modes from the analysis. The eigenfrequencies are $\omega_{k}=\sqrt{\lambda_{k}}$, and the eigenvectors are orthonormalized such that ${\vec{e}}_{k}\cdot {\vec{e}}_{l}=\sum_{i=1}^{N}{\vec{e}}_{k,i}\cdot {\vec{e}}_{l,i}=\delta_{k,l}$, where $\delta_{k,l}$ is the Kronecker delta.

In the present work, we analyze several system sizes, ranging from $N∼10^{4}$ to $10^{6}$, as in refs. 63 and 67. We compute all vibrational modes for the smallest system and only the low-frequency modes for larger systems. We then merge the mode datasets from different sizes as a function of $\omega_{k}$; results from different sizes connect smoothly, thereby extending access to the lower-frequency regime. g(ω) in Eq. **11** and $S_{\alpha }\left(q,\omega \right)$ in Eq. **16** are computed from these combined datasets, and results from different sizes are presented together in the figures.

### vDOS.

From the set of eigenfrequencies $\omega_{k}$ for vibrational modes k=1,2,…,3N, we compute the vDOS g(ω) as

[11] $$\begin{array}{c} g\left(\omega \right)=\frac{1}{3N}\sum_{k=1}^{3N}\delta \left(\omega -\omega_{k}\right), \end{array}$$

where *δ* denotes the Dirac delta function.

### Debye vDOS.

In an isotropic elastic medium, vibrational modes are phonons (1). Continuum elasticity gives the linear dispersions $\omega =c_{T}q$ and $\omega =c_{L}q$, where $c_{T}$ and $c_{L}$ are the transverse and longitudinal sound speeds, respectively. Counting phonon states yields the Debye vDOS,

[12] $$\begin{array}{c} g_{D}\left(\omega \right)=A_{D}\omega^{2}=\frac{3}{\omega_{D}^{3}}\;\omega^{2}\propto \omega^{2}, \end{array}$$

where $A_{D}=3/\omega_{D}^{3}$ is the Debye level, and the Debye frequency $\omega_{D}$ is

[13] $$\begin{array}{c} \omega_{D}={\left(\frac{c_{L}^{-3}+2c_{T}^{-3}}{3}\right)}^{-1/3}q_{D}={\left(\frac{18\pi^{2}\hat{\rho }}{c_{L}^{-3}+2c_{T}^{-3}}\right)}^{1/3}, \end{array}$$

with the Debye wavenumber $q_{D}={\left(6\pi^{2}\hat{\rho }\right)}^{1/3}$ and the number density $\hat{\rho }=N/V$.

### Stretching and Compression of Si–O Bonds In Silica Glass.

For silica glass, we quantify the stretching and compression of Si–O bonds in vibrational mode *k* via the dimensionless measure $\delta e_{k,\text{Si–O}}^{2}$, defined as

[14] $$\begin{array}{c} \delta e_{k,\text{Si–O}}^{2}=\frac{m_{\text{O}}}{N_{\text{Si–O}}}\sum_{\left\langle ij\right\rangle \in B_{\text{Si–O}}}{\left|\left(\frac{{\vec{e}}_{k,i}}{\sqrt{m_{\text{Si}}}}-\frac{{\vec{e}}_{k,j}}{\sqrt{m_{\text{O}}}}\right)\cdot {\vec{n}}_{\mathrm{ij}}\right|}^{2}, \end{array}$$

where $B_{\text{Si–O}}$ is the set of Si–O bonds (nearest-neighbor Si–O pairs), $N_{\text{Si–O}}=\left|B_{\text{Si–O}}\right|=4N_{\text{Si}}$ is the total number of Si–O bonds, as reported in Table 1, and ${\vec{n}}_{\mathrm{ij}}=\left({\vec{r}}_{i}-{\vec{r}}_{j}\right)/\left|{\vec{r}}_{i}-{\vec{r}}_{j}\right|$ is the unit vector along the Si–O bond, with *i* indexing a Si atom and *j* an O atom. This quantity captures the mean-squared relative displacement of a Si atom and its O neighbor projected along the bond direction; the mass factors render it dimensionless.

### Participation Ratio.

We measure the fraction of particles that participate in vibrational mode *k* using the participation ratio $P_{k}$ (87):

[15] $$\begin{array}{c} P_{k}=\frac{1}{N}{\left[\sum_{i=1}^{N}{\left({\vec{e}}_{k,i}\cdot {\vec{e}}_{k,i}\right)}^{2}\right]}^{-1}. \end{array}$$

For reference, $P_{k}=1$ corresponds to a perfectly extended mode with equal amplitude on all particles, whereas $P_{k}=1/N$ corresponds to a mode localized on a single particle.

### Dynamical Structure Factors.

From the data of $\omega_{k}$ and ${\vec{e}}_{k}=\left[{\vec{e}}_{k,1},{\vec{e}}_{k,2},\cdots ,{\vec{e}}_{k,N}\right]$, we compute the dynamical structure factors $S_{\alpha }\left(q,\omega \right)$, where α∈{T,L} denotes the transverse and longitudinal polarizations, respectively, following refs. 87 and 99:

[16] $$\begin{array}{c} S_{\alpha }\left(q,\omega \right)=\frac{k_{B}T}{2N}\frac{q^{2}}{\omega^{2}}\sum_{k=1}^{3N}F_{k,\alpha }\left(q\right)\delta \left(\omega -\omega_{k}\right), \end{array}$$

with

[17] $$\begin{array}{c} \begin{array}{cc} F_{k,T}\left(q\right) & ={\left|\sum_{i=1}^{N}\left(\frac{{\vec{e}}_{k,i}}{\sqrt{m_{i}}}\times \hat{\vec{q}}\right)\exp(i\vec{q}\cdot {\vec{r}}_{i})\right|}^{2},\\ F_{k,L}\left(q\right) & ={\left|\sum_{i=1}^{N}\left(\frac{{\vec{e}}_{k,i}}{\sqrt{m_{i}}}\cdot \hat{\vec{q}}\right)\exp(i\vec{q}\cdot {\vec{r}}_{i})\right|}^{2}, \end{array} \end{array}$$

where $k_{B}$ is the Boltzmann constant; $\vec{q}$ is the wavevector; $q=|\vec{q}|$ and $\hat{\vec{q}}=\vec{q}/q$; and $m_{i}$ is the mass of atom (or particle) *i*. It is important to note that scattering experiments typically access the longitudinal component $S_{L}\left(q,\omega \right)$ (100).

### Elastic Moduli and Debye Values.

We calculate the elastic moduli, the bulk modulus *K* and the shear modulus *G*, using the harmonic formulation based on the linear response theory (87, 101, 102). From these moduli, we compute Debye quantities such as the Debye frequency $\omega_{D}$ and the Debye level $A_{D}$.

Table 3 summarizes the elastic moduli and related quantities; additional data for the overconstrained-network and isostatic-network systems are provided in *SI Appendix*, Table S1. The elasticity of glasses involves not only affine but also nonaffine deformation (97, 101, 102). Consequently, an elastic modulus *M* decomposes as $M=M_{A}-M_{N}$, where $M_{A}$ and $M_{N}$ are the affine and nonaffine contributions. HS (HSH and HSL), LJ, and SS glasses exhibit strong nonaffine contributions to their shear moduli, whereas the nonaffine contribution to the bulk modulus is comparatively small; notably, in LJ and SS glasses the bulk modulus is almost entirely determined by the affine component.

**Table 3.** Physical quantities including elastic moduli and Debye values

Glass	*ρ*	*K*	$K_{A}$	$K_{N}$	$\frac{K_{N}}{K_{A}}\left(\%\right)$	*G*	$G_{A}$	$G_{N}$	$\frac{G_{N}}{G_{A}}\left(\%\right)$	*ν*	$c_{L}$	$c_{T}$	$\frac{c_{L}}{c_{T}}$	$q_{D}$	$A_{D}$	$\omega_{D}$	$\omega_{\text{BP}}$	$\omega_{0}$
Silica	2.20	40.9	172	131	76.3	30.5	104	73.6	73.7	0.202	6,090	3,720	1.64	1.58	0.00257	10.5	1.21	0.46
HSH	1.40	0.544	0.674	0.131	19.4	0.122	0.344	0.222	64.4	0.395	0.712	0.296	2.40	4.36	0.965	1.46	0.0970	0.033
HSL	1.25	0.332	0.478	0.145	30.4	0.0406	0.281	0.240	85.5	0.441	0.556	0.180	3.09	4.20	4.70	0.81	0.0379	−
LJ	1.015	61.2	61.7	0.530	0.859	13.6	35.8	22.2	61.9	0.396	8.84	3.67	2.41	3.92	0.000699	16.3	1.05	0.38
SS	1.015	40.8	40.8	0.00	0.00	6.21	14.7	8.50	57.8	0.428	6.96	2.47	2.81	3.92	0.00225	11.0	0.798	0.35

For silica glass, the quantities are measured as mass density *ρ* (g/cm^3^), elastic moduli K,G (GPa), sound speeds $c_{L},c_{T}$ (m/s), wavenumber *q* (Å^−1^), Debye level $A_{D}$ (THz^−3^), and frequency *ω* (THz). See also *SI Appendix*, Table S1 for values of the overconstrained-network system and the isostatic-network system.

By contrast, silica glass exhibits significant nonaffine contributions to both bulk and shear moduli: The nonaffine components exceed 70% of the affine components. Such strong nonaffinity in both moduli arises from the lack of centrosymmetry in the tetrahedral network structure (103, 104), which has also been observed in amorphous silicon (105) and in noncentrosymmetric crystals such as *α*-quartz (106). As a result, the bulk and shear moduli become closer in magnitude. The Poisson’s ratio ν=(3K−2G)/(6K+2G) is approximately 0.2, about half the value ν≈0.4 observed in HS, LJ, and SS glasses. Values near ν=0.2 categorize silica glass as a strong glass, whereas ν≈0.4 indicates that HS, LJ, and SS glasses are fragile glasses, as discussed in refs. 107 and 108.

## Supplementary Material

Appendix 01 (PDF)

## Data Availability

All study data are included in the article and/or *SI Appendix*. The program codes used to construct and analyze the dynamical matrices and vibrational modes have been deposited in Zenodo and are publicly available at DOI: 10.5281/zenodo.19727727 (109).

## Footnotes

^*^Throughout this paper, for silica glass, the symbol *ω* is used to denote the ordinary frequency (often written as *ν* or *f* in the literature), whereas for the other glasses (HS, LJ, and SS) *ω* denotes the angular frequency.

^†^As confirmed in refs. 63 and 67 and in *SI Appendix*, Fig. S2 for silica glass, although slight quantitative differences arise depending on the choice of the threshold value $P_{\text{th}}$, our conclusions regarding $g_{\text{EXT}}\left(\omega \right)$ and $g_{\text{QLV}}\left(\omega \right)$ remain robust: In the low-frequency regime, $g_{\text{EXT}}\left(\omega \right)$ converges to the Debye law, while $g_{\text{QLV}}\left(\omega \right)$ converges to an $\omega^{4}$ dependence and becomes gapless.

^‡^Although a quartic law $g_{\text{QLV}}\left(\omega \right)\propto \omega^{4}$ is often reported, the precise exponent remains under discussion (88–90). What matters here is that, irrespective of the exact exponent, $g_{\text{QLV}}\left(\omega \right)$ is gapless and follows a power law as ω→0. Such a gapless power-law spectrum is a hallmark of marginal stability.

^§^Below $\omega_{\text{BP}}$, we also observe modes with low $P_{k}$ indicative of localization, as seen in panels (*F* and *I*). These modes plausibly originate in regions of low local connectivity (i.e., few contacts or interacting neighbors), although a definitive identification requires further study.

## References

[1] N. W. Ashcroft, N. D. Mermin, Solid State Physics (Harcourt College Publishers, New York, 1976).

[2] M. A. Ramos, Low-Temperature Thermal and Vibrational Properties of Disordered Solids: A Half-Century of Universal “Anomalies” of Glasses (World Scientific Pub Co Inc., 2022).

[3] T. Nakayama, Boson peak and terahertz frequency dynamics of vitreous silica. Rep. Prog. Phys. **65**, 1195 (2002).

[4] U. Buchenau , Low-frequency modes in vitreous silica. Phys. Rev. B **34**, 5665–5673 (1986). 10.1103/physrevb.34.5665 9940402

[5] O. Yamamuro , Inelastic neutron scattering study of low energy excitations in glassy 1-butene. J. Chem. Phys. **105**, 732–737 (1996).

[6] M. Arai , Novel existence of collective propagating mode and strongly localized mode in vitreous silica. Phys. B Condens. Matter 263–264, 268–272 (1999).

[7] M. J. Harris, M. T. Dove, J. M. Parker, Floppy modes and the boson peak in crystalline and amorphous silicates: An inelastic neutron scattering study. Mineral. Mag. **64**, 435–440 (2000).

[8] A. Monaco , Effect of densification on the density of vibrational states of glasses. Phys. Rev. Lett. **97**, 135501 (2006). 17026042 10.1103/PhysRevLett.97.135501

[9] K. Niss , Influence of pressure on the boson peak: Stronger than elastic medium transformation. Phys. Rev. Lett. **99**, 055502 (2007). 17930767 10.1103/PhysRevLett.99.055502

[10] B. Rufflé, D. A. Parshin, E. Courtens, R. Vacher, Boson peak and its relation to acoustic attenuation in glasses. Phys. Rev. Lett. **100**, 015501 (2008). 18232782 10.1103/PhysRevLett.100.015501

[11] G. Baldi , Thermal conductivity and terahertz vibrational dynamics of vitreous silica. Phys. Rev. B **77**, 214309 (2008).

[12] G. Monaco, V. M. Giordano, Breakdown of the debye approximation for the acoustic modes with nanometric wavelengths in glasses. Proc. Natl. Acad. Sci. U.S.A. **106**, 3659 (2009). 19240211 10.1073/pnas.0808965106 PMC2656136

[13] G. Baldi, V. M. Giordano, G. Monaco, B. Ruta, Sound attenuation at terahertz frequencies and the boson peak of vitreous silica. Phys. Rev. Lett. **104**, 195501 (2010). 20866974 10.1103/PhysRevLett.104.195501

[14] G. Baldi, V. M. Giordano, G. Monaco, Elastic anomalies at terahertz frequencies and excess density of vibrational states in silica glass. Phys. Rev. B **83**, 174203 (2011).

[15] B. Ruta , Acoustic excitations in glassy sorbitol and their relation with the fragility and the boson peak. J. Chem. Phys. **137**, 214502 (2012). 23231246 10.1063/1.4768955

[16] G. Baldi , Emergence of crystal-like atomic dynamics in glasses at the nanometer scale. Phys. Rev. Lett. **110**, 185503 (2013). 23683216 10.1103/PhysRevLett.110.185503

[17] A. I. Chumakov , Role of disorder in the thermodynamics and atomic dynamics of glasses. Phys. Rev. Lett. **112**, 025502 (2014). 24484025 10.1103/PhysRevLett.112.025502

[18] G. Baldi, V. M. Giordano, B. Ruta, G. Monaco, On the nontrivial wave-vector dependence of the elastic modulus of glasses. Phys. Rev. B **93**, 144204 (2016).

[19] R. C. Zeller, R. O. Pohl, Thermal conductivity and specific heat of noncrystalline solids. Phys. Rev. B **4**, 2029–2041 (1971).

[20] P. W. Anderson, B. I. Halperin, C. M. Varma, Anomalous low-temperature thermal properties of glasses and spin glasses. Philos. Mag. **25**, 1–9 (1972).

[21] D. Cahill, R. O. Pohl, Lattice vibrations and heat transport in crystals and glasses. Annu. Rev. Phys. Chem. **39**, 93–121 (1988).

[22] G. Monaco, S. Mossa, Anomalous properties of the acoustic excitations in glasses on the mesoscopic length scale. Proc. Natl. Acad. Sci. U.S.A. **106**, 16907 (2009). 19805115 10.1073/pnas.0903922106 PMC2761319

[23] H. Mizuno, S. Mossa, J. L. Barrat, Acoustic excitations and elastic heterogeneities in disordered solids. Proc. Natl. Acad. Sci. U.S.A. **111**, 11949–11954 (2014). 25092324 10.1073/pnas.1409490111 PMC4143046

[24] A. Tanguy, Vibrations and heat transfer in glasses: The role played by disorder. Comptes Rendus. Phys. **24**, 73–97 (2023).

[25] F. Leonforte, R. Boissière, A. Tanguy, J. P. Wittmer, J. L. Barrat, Continuum limit of amorphous elastic bodies. III. Three-dimensional systems. Phys. Rev. B **72**, 224206 (2005).

[26] F. Léonforte, A. Tanguy, J. P. Wittmer, J. L. Barrat, Inhomogeneous elastic response of silica glass. Phys. Rev. Lett. **97**, 055501 (2006). 17026110 10.1103/PhysRevLett.97.055501

[27] A. Tanguy, B. Mantisi, M. Tsamados, Vibrational modes as a predictor for plasticity in a model glass. Eur. Lett. **90**, 16004 (2010).

[28] M. L. Manning, A. J. Liu, Vibrational modes identify soft spots in a sheared disordered packing. Phys. Rev. Lett. **107**, 108302 (2011). 21981537 10.1103/PhysRevLett.107.108302

[29] W. Schirmacher, Thermal conductivity of glassy materials and the “boson peak”. Eur. Lett. **73**, 892 (2006).

[30] A. Marruzzo, W. Schirmacher, A. Fratalocchi, G. Ruocco, Heterogeneous shear elasticity of glasses: The origin of the boson peak. Sci. Rep. **3**, 1407 (2013). 23470597 10.1038/srep01407 PMC3591752

[31] H. Mizuno, S. Mossa, J. L. Barrat, Elastic heterogeneity, vibrational states, and thermal conductivity across an amorphisation transition. EPL **104**, 56001 (2013).

[32] W. Schirmacher, T. Scopigno, G. Ruocco, Theory of vibrational anomalies in glasses. J. Noncryst. Solids **407**, 133–140 (2015).

[33] L. Zhang , Experimental studies of vibrational modes in a two-dimensional amorphous solid. Nat. Commun. **8**, 67 (2017). 28694525 10.1038/s41467-017-00106-5 PMC5503991

[34] V. G. Karpov, M. I. Klinger, F. N. Ignat’ev, Theory of the low-temperature anomalies in the thermal properties of amorphous structures. Sov. Phys. JETP **57**, 439 (1983).

[35] U. Buchenau, Y. M. Galperin, V. L. Gurevich, H. R. Schober, Anharmonic potentials and vibrational localization in glasses. Phys. Rev. B **43**, 5039–5045 (1991). 10.1103/physrevb.43.5039 9997881

[36] V. L. Gurevich, D. A. Parshin, H. R. Schober, Anharmonicity, vibrational instability, and the boson peak in glasses. Phys. Rev. B **67**, 094203 (2003).

[37] E. Bouchbinder, E. Lerner, C. Rainone, P. Urbani, F. Zamponi, Low-frequency vibrational spectrum of mean-field disordered systems. Phys. Rev. B **103**, 174202 (2021).

[38] Y. M. Beltukov, V. I. Kozub, D. A. Parshin, Ioffe-regel criterion and diffusion of vibrations in random lattices. Phys. Rev. B **87**, 134203 (2013).

[39] F. Vogel, M. Fuchs, Vibrational phenomena in glasses at low temperatures captured by field theory of disordered harmonic oscillators. Phys. Rev. Lett. **130**, 236101 (2023). 37354405 10.1103/PhysRevLett.130.236101

[40] F. Vogel, P. Baumgärtel, M. Fuchs, Self-consistent current response theory of unjamming and vibrational modes in low-temperature amorphous solids. Phys. Rev. X **15**, 011030 (2025).

[41] J. C. Phillips, Topology of covalent non-crystalline solids I: Short-range order in chalcogenide alloys. J. Noncrystal. Solids **34**, 153–181 (1979).

[42] G. H. Döhler, R. Dandoloff, H. Bilz, A topological-dynamical model of amorphycity. J. Noncrystal. Solids **42**, 87–95 (1980).

[43] J. C. Phillips, Topology of covalent non-crystalline solids II: Medium-range order in chalcogenide alloys and A-Si(Ge). J. Noncrystal. Solids **43**, 37–77 (1981).

[44] M. F. Thorpe, Continuous deformations in random networks. J. Noncrystal. Solids **57**, 355–370 (1983).

[45] J. C. Maxwell, L., on the calculation of the equilibrium and stiffness of frames. Lond. Edinb. Dublin Philos. Mag. J. Sci. **27**, 294–299 (1864).

[46] S. Feng, M. F. Thorpe, E. Garboczi, Effective-medium theory of percolation on central-force elastic networks. Phys. Rev. B **31**, 276–280 (1985). 10.1103/physrevb.31.276 9935421

[47] H. He, M. F. Thorpe, Elastic properties of glasses. Phys. Rev. Lett. **54**, 2107–2110 (1985). 10031231 10.1103/PhysRevLett.54.2107

[48] Y. Cai, M. F. Thorpe, Floppy modes in network glasses. Phys. Rev. B **40**, 10535–10542 (1989). 10.1103/physrevb.40.10535 9991603

[49] D. J. Jacobs, M. F. Thorpe, Generic rigidity percolation: The pebble game. Phys. Rev. Lett. **75**, 4051–4054 (1995). 10059802 10.1103/PhysRevLett.75.4051

[50] K. Trachenko, M. T. Dove, K. D. Hammonds, M. J. Harris, V. Heine, Low energy dynamics and tunneling states in silica glass. Phys. Rev. Lett. **81**, 3431–3434 (1998).

[51] K. O. Trachenko, M. T. Dove, M. J. Harris, V. Heine, Dynamics of silica glass: two-level tunnelling states and low-energy floppy modes. J. Phys. Condens. Matter **12**, 8041 (2000).

[52] D. J. Durian, Foam mechanics at the bubble scale. Phys. Rev. Lett. **75**, 4780–4783 (1995). 10059995 10.1103/PhysRevLett.75.4780

[53] C. S. O’Hern, L. E. Silbert, A. J. Liu, S. R. Nagel, Jamming at zero temperature and zero applied stress: The epitome of disorder. Phys. Rev. E **68**, 011306 (2003). 10.1103/PhysRevE.68.011306 12935136

[54] M. van Hecke, Jamming of soft particles: Geometry, mechanics, scaling and isostaticity. J. Phys. Condens. Matter **22**, 033101 (2009). 21386274 10.1088/0953-8984/22/3/033101

[55] M. Wyart, On the rigidity of amorphous solids. Ann. Phys. **30**, 1–96 (2005).

[56] M. Wyart, S. R. Nagel, T. A. Witten, Geometric origin of excess low-frequency vibrational modes in weakly connected amorphous solids. EPL **72**, 486 (2005).

[57] M. Wyart, L. E. Silbert, S. R. Nagel, T. A. Witten, Effects of compression on the vibrational modes of marginally jammed solids. Phys. Rev. E **72**, 051306 (2005). 10.1103/PhysRevE.72.051306 16383602

[58] M. Wyart, Scaling of phononic transport with connectivity in amorphous solids. EPL **89**, 64001 (2010).

[59] E. DeGiuli, A. Laversanne-Finot, G. Düring, E. Lerner, M. Wyart, Effects of coordination and pressure on sound attenuation, boson peak and elasticity in amorphous solids. Soft Matter **10**, 5628–5644 (2014). 24981002 10.1039/c4sm00561a

[60] L. E. Silbert, A. J. Liu, S. R. Nagel, Vibrations and diverging length scales near the unjamming transition. Phys. Rev. Lett. **95**, 098301 (2005). 16197259 10.1103/PhysRevLett.95.098301

[61] N. Xu, V. Vitelli, A. J. Liu, S. R. Nagel, Anharmonic and quasi-localized vibrations in jammed solids - modes for mechanical failure. Europhys. Lett. **90**, 56001 (2010).

[62] P. Charbonneau, E. I. Corwin, G. Parisi, A. Poncet, F. Zamponi, Universal non-debye scaling in the density of states of amorphous solids. Phys. Rev. Lett. **117**, 045503 (2016). 27494482 10.1103/PhysRevLett.117.045503

[63] H. Mizuno, H. Shiba, A. Ikeda, Continuum limit of the vibrational properties of amorphous solids. Proc. Natl. Acad. Sci. U.S.A. **114**, E9767–E9774 (2017). 29087941 10.1073/pnas.1709015114 PMC5699054

[64] H. Mizuno, A. Ikeda, Phonon transport and vibrational excitations in amorphous solids. Phys. Rev. E **98**, 062612 (2018).

[65] M. Shimada, H. Mizuno, L. Berthier, A. Ikeda, Low-frequency vibrations of jammed packings in large spatial dimensions. Phys. Rev. E **101**, 052906 (2020). 32575185 10.1103/PhysRevE.101.052906

[66] N. Xu, M. Wyart, A. J. Liu, S. R. Nagel, Excess vibrational modes and the boson peak in model glasses. Phys. Rev. Lett. **98**, 175502 (2007).

[67] M. Shimada, H. Mizuno, A. Ikeda, Anomalous vibrational properties in the continuum limit of glasses. Phys. Rev. E **97**, 022609 (2018). 29548203 10.1103/PhysRevE.97.022609

[68] L. Wang , Low-frequency vibrational modes of stable glasses. Nat. Commun. **10**, 26 (2019). 30604770 10.1038/s41467-018-07978-1 PMC6318266

[69] C. Brito, M. Wyart, On the rigidity of a hard-sphere glass near random close packing. Europhys. Lett. **76**, 149 (2006).

[70] C. Brito, M. Wyart, Geometric interpretation of previtrification in hard sphere liquids. J. Chem. Phys. **131**, 024504 (2009). 19604001 10.1063/1.3157261

[71] E. Lerner, E. Bouchbinder, Frustration-induced internal stresses are responsible for quasilocalized modes in structural glasses. Phys. Rev. E **97**, 032140 (2018). 29776173 10.1103/PhysRevE.97.032140

[72] M. Shimada, H. Mizuno, M. Wyart, A. Ikeda, Spatial structure of quasilocalized vibrations in nearly jammed amorphous solids. Phys. Rev. E **98**, 060901 (2018).

[73] S. N. Taraskin, S. R. Elliott, Nature of vibrational excitations in vitreous silica. Phys. Rev. B **56**, 8605–8622 (1997).

[74] D. Richard , Universality of the nonphononic vibrational spectrum across different classes of computer glasses. Phys. Rev. Lett. **125**, 085502 (2020). 32909789 10.1103/PhysRevLett.125.085502

[75] N. S. Shcheblanov, M. E. Povarnitsyn, J. D. Wiles, S. R. Elliott, S. N. Taraskin, Quasilocalized vibrations in vitreous silica. Phys. Status Solidi **258**, 2000422 (2021).

[76] R. Guerra, S. Bonfanti, I. Procaccia, S. Zapperi, Universal density of low-frequency states in silica glass at finite temperatures. Phys. Rev. E **105**, 054104 (2022). 35706171 10.1103/PhysRevE.105.054104

[77] S. Sundararaman, L. Huang, S. Ispas, W. Kob, New optimization scheme to obtain interaction potentials for oxide glasses. J. Chem. Phys. **148**, 194504 (2018). 30307185 10.1063/1.5023707

[78] B. W. H. van Beest, G. J. Kramer, R. A. van Santen, Force fields for silicas and aluminophosphates based on ab initio calculations. Phys. Rev. Lett. **64**, 1955–1958 (1990). 10041537 10.1103/PhysRevLett.64.1955

[79] D. Wolf, Reconstruction of nacl surfaces from a dipolar solution to the madelung problem. Phys. Rev. Lett. **68**, 3315–3318 (1992). 10045671 10.1103/PhysRevLett.68.3315

[80] D. Wolf, P. Keblinski, S. R. Phillpot, J. Eggebrecht, Exact method for the simulation of coulombic systems by spherically truncated, pairwise r-1 summation. J. Chem. Phys. **110**, 8254–8282 (1999).

[81] A. Carré, L. Berthier, J. Horbach, S. Ispas, W. Kob, Amorphous silica modeled with truncated and screened coulomb interactions: A molecular dynamics simulation study. J. Chem. Phys. **127**, 114512 (2007). 17887862 10.1063/1.2777136

[82] A. Carré, S. Ispas, J. Horbach, W. Kob, Developing empirical potentials from ab initio simulations: The case of amorphous silica. Comput. Mater. Sci. **124**, 323–334 (2016).

[83] S. Nosé, A unified formulation of the constant temperature molecular dynamics methods. J. Chem. Phys. **81**, 511–519 (1984).

[84] W. G. Hoover, Canonical dynamics: Equilibrium phase-space distributions. Phys. Rev. A **31**, 1695–1697 (1985). 10.1103/physreva.31.1695 9895674

[85] T. Damart, A. Tanguy, D. Rodney, Theory of harmonic dissipation in disordered solids. Phys. Rev. B **95**, 054203 (2017).

[86] A. El Hamdaoui, E. M. Ghardi, M. J. D. Rushton, A. Hasnaoui, S. Ouaskit, The impact of densification on the boson peak and structure in vitreous silica. J. Am. Ceram. Soc. **108**, e20631 (2025).

[87] H. Mizuno, A. Ikeda, *Computational Simulations of the Vibrational Properties of Glasses*, M. A. Ramos, Ed. (World Scientific, Europe, 2022), pp. 375–433.

[88] W. Schirmacher , The nature of non-phononic excitations in disordered systems. Nat. Commun. **15**, 3107 (2024). 38600083 10.1038/s41467-024-46981-7 PMC11258284

[89] D. Xu, S. Zhang, H. Tong, L. Wang, N. Xu, Low-frequency vibrational density of states of ordinary and ultra-stable glasses. Nat. Commun. **15**, 1424 (2024). 38365816 10.1038/s41467-024-45671-8 PMC11258317

[90] E. Lerner, E. Bouchbinder, Testing the heterogeneous-elasticity theory for low-energy excitations in structural glasses. Phys. Rev. E **111**, L013402 (2025). 39972860 10.1103/PhysRevE.111.L013402

[91] Y. C. Hu, H. Tanaka, Origin of the boson peak in amorphous solids. Nat. Phys. **18**, 669–677 (2022).

[92] S. Mahajan , A flat-mode perspective on the boson peak in amorphous solids. arXiv [Preprint] (2025). http://arxiv.org/abs/2509.06340 (Accessed 5 December 2025).

[93] P. Vashishta, R. K. Kalia, J. P. Rino, I. Ebbsjö, Interaction potential for sio2: A molecular-dynamics study of structural correlations. Phys. Rev. B **41**, 12197–12209 (1990). 10.1103/physrevb.41.12197 9993674

[94] E. Lerner, E. DeGiuli, G. During, M. Wyart, Breakdown of continuum elasticity in amorphous solids. Soft Matter **10**, 5085–5092 (2014). 24905568 10.1039/c4sm00311j

[95] K. Karimi, C. E. Maloney, Elasticity of frictionless particles near jamming. Phys. Rev. E **92**, 022208 (2015). 10.1103/PhysRevE.92.022208 26382395

[96] D. Hexner, A. J. Liu, S. R. Nagel, Two diverging length scales in the structure of jammed packings. Phys. Rev. Lett. **121**, 115501 (2018). 30265103 10.1103/PhysRevLett.121.115501

[97] H. Mizuno, S. Mossa, J. L. Barrat, Measuring spatial distribution of the local elastic modulus in glasses. Phys. Rev. E **87**, 042306 (2013). 10.1103/PhysRevE.87.042306 23679413

[98] H. Mizuno, L. E. Silbert, M. Sperl, S. Mossa, J. L. Barrat, Cutoff nonlinearities in the low-temperature vibrations of glasses and crystals. Phys. Rev. E **93**, 043314 (2016). 27176435 10.1103/PhysRevE.93.043314

[99] G. S. Grest, S. R. Nagel, A. Rahman, Longitudinal and transverse excitations in a glass. Phys. Rev. Lett. **49**, 1271–1274 (1982).

[100] J. B. Suck, P. A. Egelstaff, R. A. Robinson, D. S. Sivia, A. D. Taylor, Brillouin scattering with neutrons in an amorphous solid. Europhys. Lett. **19**, 207 (1992).

[101] A. Lemaitre, C. Maloney, Sum rules for the quasi-static and visco-elastic response of disordered solids at zero temperature. J. Stat. Phys. **123**, 415–453 (2006).

[102] H. Mizuno, K. Saitoh, L. E. Silbert, Elastic moduli and vibrational modes in jammed particulate packings. Phys. Rev. E **93**, 062905 (2016). 27415345 10.1103/PhysRevE.93.062905

[103] R. Milkus, A. Zaccone, Local inversion-symmetry breaking controls the boson peak in glasses and crystals. Phys. Rev. B **93**, 094204 (2016).

[104] J. Krausser, R. Milkus, A. Zaccone, Non-affine lattice dynamics of defective fcc crystals. Soft Matter **13**, 6079–6089 (2017). 28785752 10.1039/c7sm00843k

[105] E. Minamitani, T. Nakamura, I. Obayashi, H. Mizuno, Persistent homology elucidates hierarchical structures responsible for mechanical properties in covalent amorphous solids. Nat. Commun. **16**, 8226 (2025). 40998805 10.1038/s41467-025-63424-z PMC12462471

[106] B. Cui, A. Zaccone, D. Rodney, Nonaffine lattice dynamics with the Ewald method reveals strongly nonaffine elasticity of *α*-quartz. J. Chem. Phys. **151**, 224509 (2019). 31837668 10.1063/1.5129025

[107] G. N. Greaves, A. L. Greer, R. S. Lakes, T. Rouxel, Poisson’s ratio and modern materials. Nat. Mater. **10**, 823–837 (2011). 22020006 10.1038/nmat3134

[108] E. Duval, T. Deschamps, L. Saviot, Poisson ratio and excess low-frequency vibrational states in glasses. J. Chem. Phys. **139**, 064506 (2013). 23947870 10.1063/1.4817778

[109] H. Mizuno, Vibrational analysis codes for “Boson peak in covalent network glasses: Isostaticity and marginal stability.” Zenodo. 10.5281/zenodo.19727727. Deposited 25 April 2026. PMC13229216 42213760

