# Supplementary material for: Boson peak in covalent network glasses: Isostaticity and marginal stability
Source: Proc Natl Acad Sci U S A. 2026 May 29;123(22):e2528998123. doi: 10.1073/pnas.2528998123 (PMC13229216; doi:10.1073/pnas.2528998123)
Supplement: Supplementary file 1 — Appendix 01 (PDF) [file pnas.2528998123.sapp.pdf]

Supporting Information for:

Boson peak in covalent network glasses: Isostaticity and marginal stability

by Hideyuki Mizuno, Tatsuya Mori, Giacomo Baldi, and Emi Minamitani

Email: hideyuki.mizuno@phys.c.u-tokyo.ac.jp

I. SUPPLEMENTARY DATA

In the following, we report supplementary data, including vibrational states in HSL and SS glasses (Fig. S1), dependence of the vDOS on the threshold value of the participation ratio in silica glass (Fig. S2), dynamical structure factors of the isostatic-network system of silica glass (Fig. S3), transverse dynamical structure factor of HSL and SS glasses (Fig. S4), longitudinal dynamical structure factor of HSH, LJ, HSL, and SS glasses (Fig. S5), and physical quantities including elastic moduli and Debye values (Table S1).

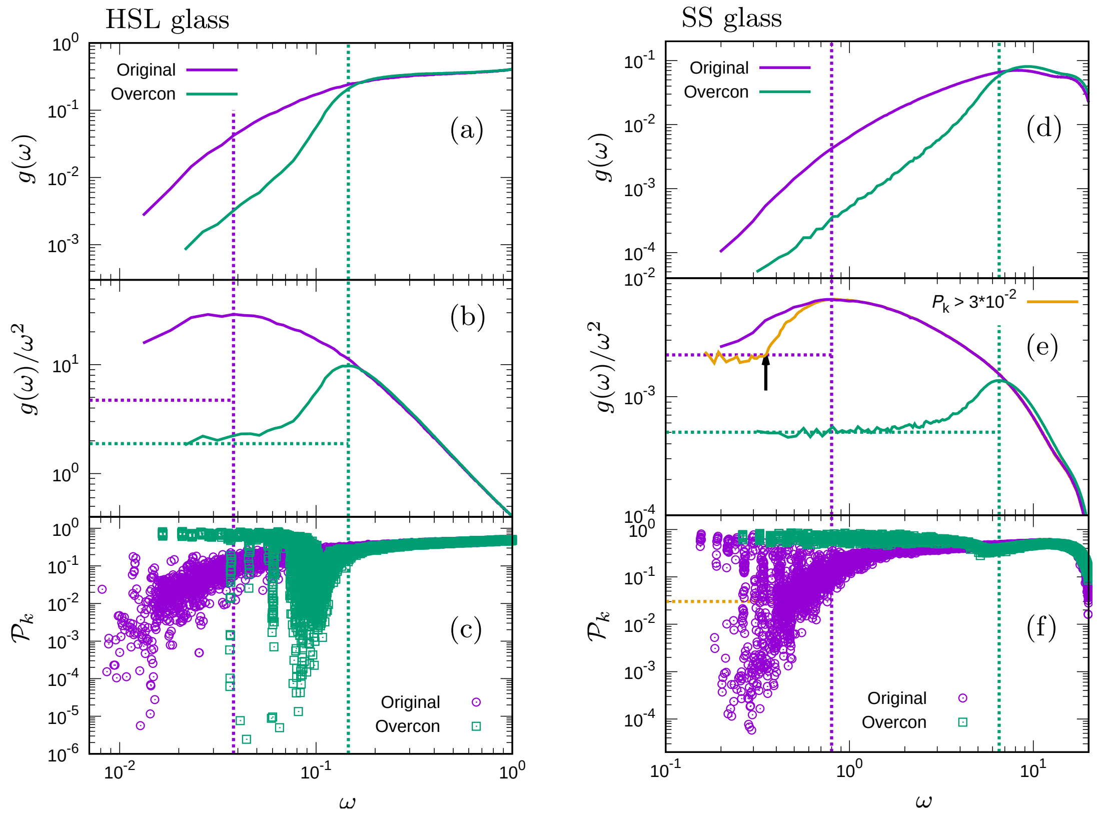

FIG. S1. Vibrational states in the low-frequency regime in HSL and SS glasses. (a–c) HSL glass and (d–f) SS glass. $g(\omega)$, $g(\omega)/\omega^2$, and $\mathcal{P}_k$ are plotted as functions of frequency ω for the original system (purple) and the overconstrained-network system (green). The vertical lines mark the BP frequency ω_{BP} for the original and overconstrained-network systems, while the horizontal dotted lines in (b,e) indicate the Debye level A_D. In addition, panel (e) for SS glass shows, in orange, the vDOS $g_{\mathrm{EXT}}(\omega)$ of extended modes with $\mathcal{P}_k > \mathcal{P}_{\mathrm{th}} = 3 \times 10^{-2}$. This threshold $\mathcal{P}_{\mathrm{th}}$ is indicated by the horizontal dotted line in (f). The arrow in (e) marks the continuum-limit frequency ω_0 at which $g_{\mathrm{EXT}}(\omega)/\omega^2$ converges to A_D.

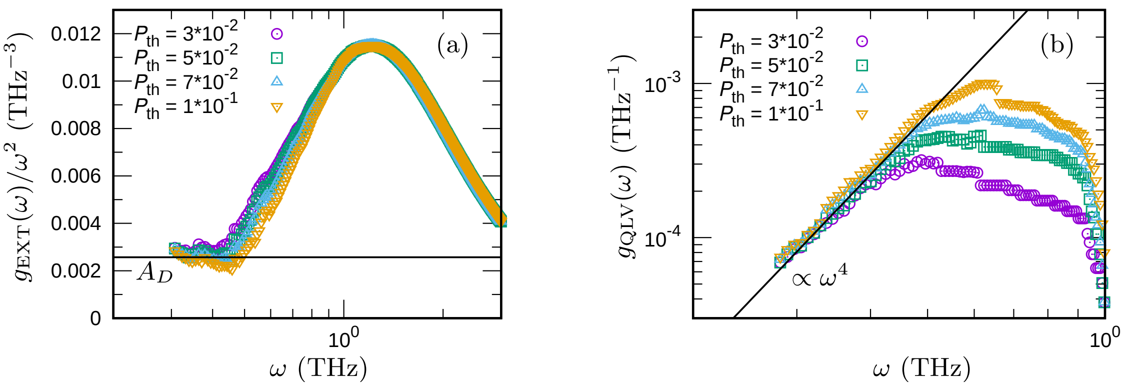

FIG. S2. Dependence of the vDOS on the threshold value of the participation ratio in silica glass. Panels (a) and (b) respectively show $g_{\mathrm{EXT}}(\omega)$ for modes with $\mathcal{P}_k > \mathcal{P}_{\mathrm{th}}$ and $g_{\mathrm{QLV}}(\omega)$ for modes with $\mathcal{P}_k \leq \mathcal{P}_{\mathrm{th}}$. The threshold value $\mathcal{P}_{\mathrm{th}}$ is varied from 3×10^{-2} to 1×10^{-1}. Although slight quantitative differences arise depending on the choice of $\mathcal{P}_{\mathrm{th}}$, our conclusions regarding $g_{\mathrm{EXT}}(\omega)$ and $g_{\mathrm{QLV}}(\omega)$ remain robust: in the low-frequency regime, $g_{\mathrm{EXT}}(\omega)$ converges to the Debye law $A_D\omega^2$, while $g_{\mathrm{QLV}}(\omega)$ converges to an ω^4 dependence and becomes gapless.

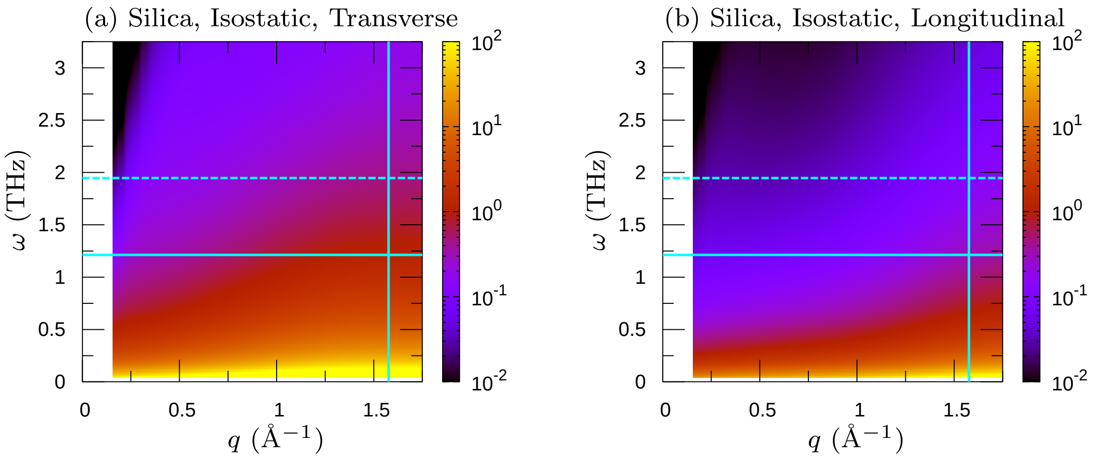

FIG. S3. Dynamical structure factors of the isostatic-network system of silica glass. Panels (a) and (b) show the transverse $S_T(q,\omega)/(k_BT)$ and longitudinal $S_L(q,\omega)/(k_BT)$, respectively, as functions of q and ω. Values are plotted in units of $(\mathrm{eV\,THz})^{-1}$. The vertical line marks the Debye wavenumber q_D. Horizontal solid and dotted lines indicate, for reference, the BP frequency ω_{BP} of the original system and the overconstrained-network system, respectively. Note that $\omega_{\mathrm{BP}} \to 0$ in the isostatic-network system.

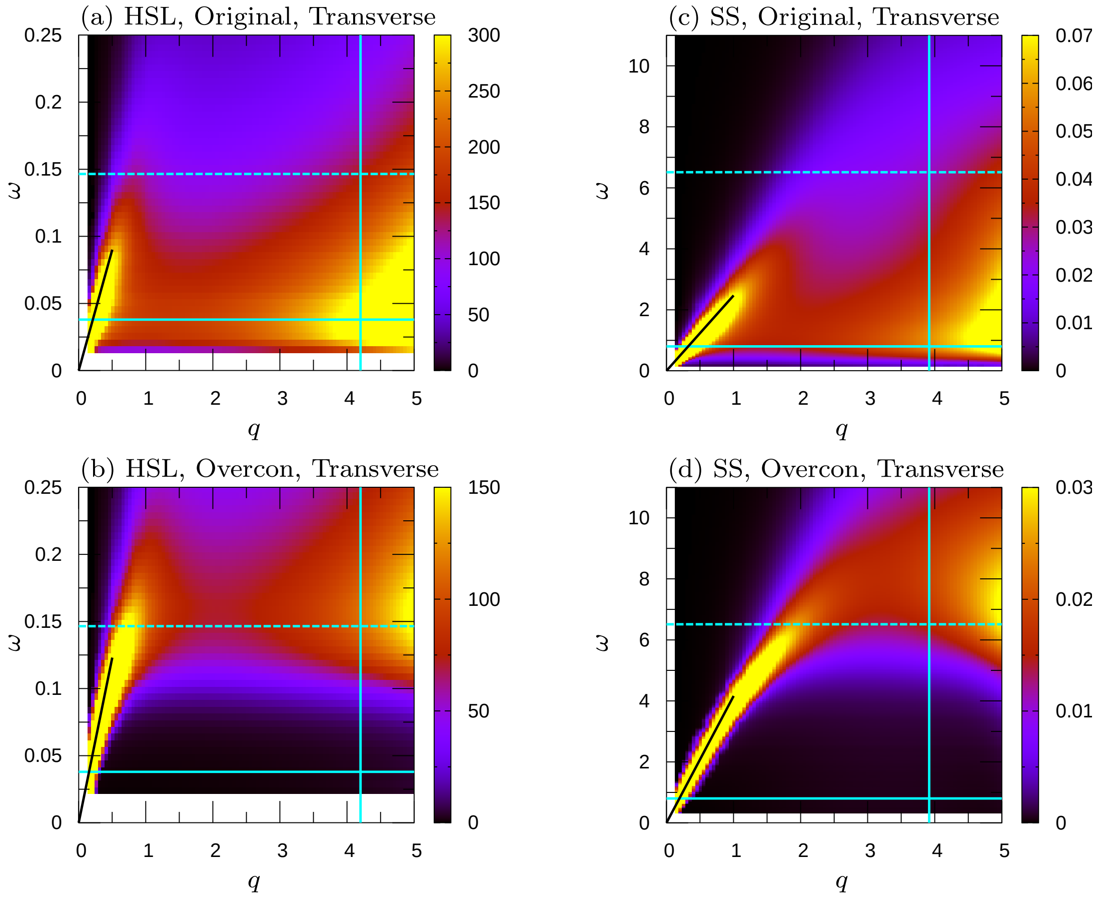

FIG. S4. Transverse dynamical structure factor of HSL and SS glasses. (a,b) HSL glass and (c,d) SS glass. $S_T(q,\omega)/(k_BT)$ is shown as a function of q and ω for the original systems in (a,c) and the overconstrained-network systems in (b,d). The vertical line marks the Debye wavenumber q_D. Horizontal solid and dotted lines indicate the BP frequency ω_{BP} for the original and overconstrained-network systems, respectively. The black solid curve shows the linear dispersion $\omega = c_T q$, with c_T being the transverse sound speed, corresponding to phonon excitations.

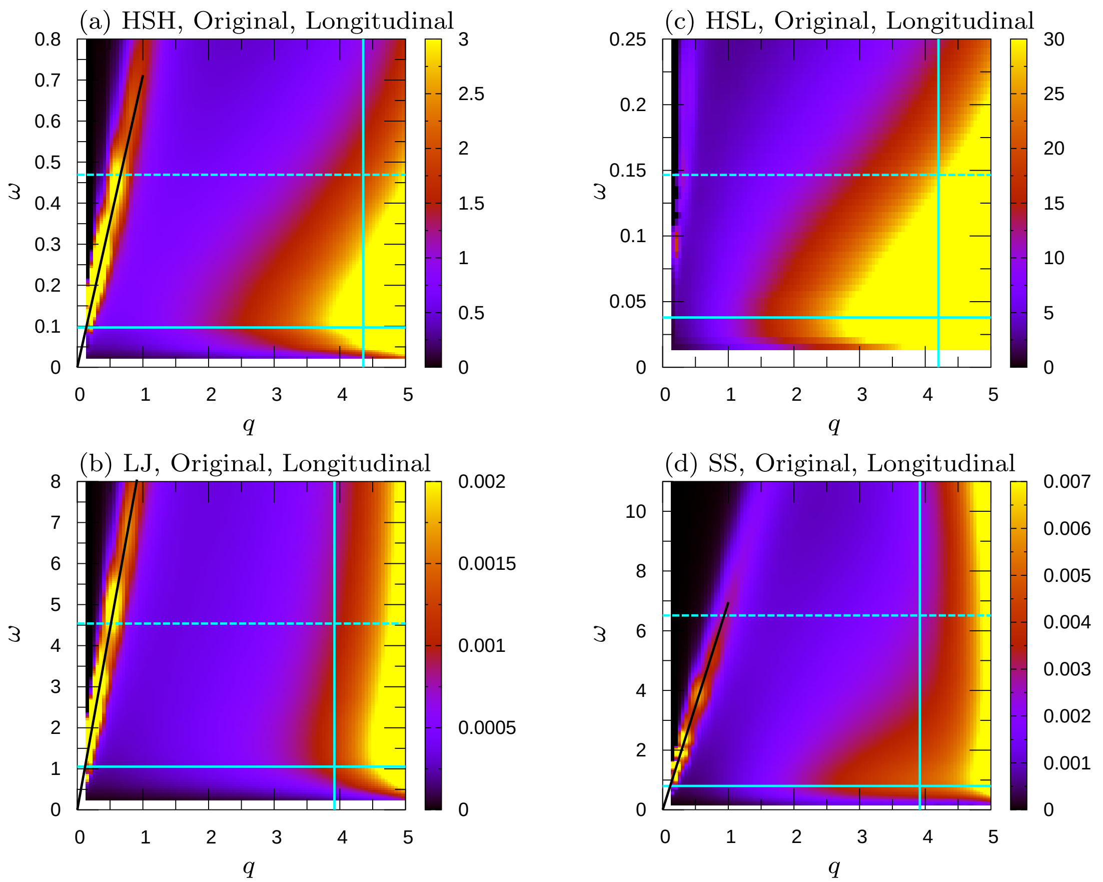

FIG. S5. Longitudinal dynamical structure factor of HSH, LJ, HSL, and SS glasses. (a) HSH glass, (b) LJ glass, (c) HSL glass, and (d) SS glass. $S_L(q,\omega)/(k_BT)$ is shown as a function of q and ω for the original systems. The vertical line marks the Debye wavenumber q_D. Horizontal solid and dotted lines indicate, for reference, the BP frequency ω_{BP} for the original and overconstrained-network systems, respectively. The black solid curve shows the linear dispersion $\omega = c_L q$, with c_L being the longitudinal sound speed, corresponding to phonon excitations.

TABLE S1. Physical quantities including elastic moduli and Debye values. For silica glass, the quantities are measured as mass density ρ (g/cm^3), elastic moduli K, G (GPa), sound speeds c_L, c_T (m/s), wavenumber q (Å^{-1}), Debye level A_D (THz^{-3}), and frequency ω (THz). In addition to values of the original system (reported in Table 3 of the main text), we list results for the overconstrained-network system and the isostatic-network system.

		ρ	K	K_A	K_N	$\frac{K_N}{K_A}$ (%)	G	G_A	G_N	$\frac{G_N}{G_A}$ (%)	ν	c_L	c_T	$\frac{c_L}{c_T}$	q_D	A_D	ω_D	ω_{BP}
Silica	Original	2.20	40.9	172	131	76.3	30.5	104	73.6	73.7	0.202	6090	3720	1.64	1.58	0.00257	10.5	1.21
	Overcon	2.20	53.6	203	149	73.6	29.4	121	92.4	75.8	0.268	6500	3660	1.78	1.58	0.00265	10.4	1.95
	Isostatic	2.20	0	196	196	100	0	118	118	100	–	0	0	–	1.58	$+\infty$	0	0
HSH	Original	1.40	0.544	0.674	0.131	19.4	0.122	0.344	0.222	64.4	0.395	0.712	0.296	2.40	4.36	0.965	1.46	0.0970
	Overcon	1.40	0.548	0.641	0.0932	14.4	0.246	0.385	0.138	36.0	0.305	0.793	0.420	1.89	4.36	0.351	2.04	0.469
HSL	Original	1.25	0.332	0.478	0.145	30.4	0.0406	0.281	0.240	85.5	0.441	0.556	0.180	3.09	4.20	4.70	0.81	0.0379
	Overcon	1.25	0.347	0.475	0.128	26.9	0.0757	0.285	0.209	73.4	0.398	0.598	0.246	2.43	4.20	1.88	1.17	0.146
LJ	Original	1.015	61.2	61.7	0.530	0.859	13.6	35.8	22.2	61.9	0.396	8.84	3.67	2.41	3.92	0.000699	16.3	1.05
	Overcon	1.015	60.6	61.0	0.415	0.680	21.6	36.6	15.0	41.1	0.341	9.39	4.61	2.04	3.92	0.000360	20.3	4.54
SS	Original	1.015	40.8	40.8	0.00	0.00	6.21	14.7	8.50	57.8	0.428	6.96	2.47	2.81	3.92	0.00225	11.0	0.798
	Overcon	1.015	35.4	35.4	0.00	0.00	17.6	21.2	3.66	17.2	0.287	7.61	4.16	1.83	3.92	0.000499	18.2	6.51

II. DERIVATION OF EQUATION (2) IN THE MAIN PAPER

The dynamical structure factors $S_\alpha(q,\omega)$, where $\alpha \in \{T, L\}$ denotes the transverse and longitudinal polarizations, respectively, are defined as follows [1, 2]:

$$S_\alpha(q,\omega) = \frac{k_B T}{2N}\frac{q^2}{\omega^2}\sum_{k=1}^{3N} F_{k,\alpha}(q)\,\delta(\omega-\omega_k)\,, \tag{S1}$$

with

$$\begin{aligned} F_{k,T}(q) &= \left|\sum_{i=1}^{N}\left(\frac{\vec{e}_{k,i}}{\sqrt{m_i}}\times\hat{\vec{q}}\right)\exp\!\left(\mathrm{i}\vec{q}\cdot\vec{r}_i\right)\right|^2, \\ F_{k,L}(q) &= \left|\sum_{i=1}^{N}\left(\frac{\vec{e}_{k,i}}{\sqrt{m_i}}\cdot\hat{\vec{q}}\right)\exp\!\left(\mathrm{i}\vec{q}\cdot\vec{r}_i\right)\right|^2, \end{aligned} \tag{S2}$$

where k_B is the Boltzmann constant; $q = |\vec{q}|$ and ω are the wavenumber and frequency, respectively; $\hat{\vec{q}} = \vec{q}/q$ is the unit vector along $\vec{q}$; and m_i and $\vec{e}_{k,i}$ are the mass and the eigenvector of atom (or particle) i, respectively.

Using Eqs. (S1) and (S2), we obtain

$$\int_0^{q_D}\left\{\frac{S_T(q,\omega)}{k_BT}+\frac{S_L(q,\omega)}{k_BT}\right\}dq = \frac{1}{2N\omega^2}\sum_{k=1}^{3N}\delta(\omega-\omega_k)\left(\sum_{i,j=1}^{N}\frac{\vec{e}_{k,i}\cdot\vec{e}_{k,j}}{\sqrt{m_im_j}}\int_0^{q_D}e^{\mathrm{i}\vec{q}\cdot(\vec{r}_i-\vec{r}_j)}q^2\,dq\right). \tag{S3}$$

Assuming an isotropic elastic medium, we have

$$\int_0^{q_D}e^{\mathrm{i}\vec{q}\cdot(\vec{r}_i-\vec{r}_j)}q^2\,dq = \frac{1}{4\pi}\int_{0\le|\vec{q}|\le q_D}e^{\mathrm{i}\vec{q}\cdot(\vec{r}_i-\vec{r}_j)}\,d^3\vec{q} = \frac{1}{4\pi}\delta_{i,j}\left(\int_{0\le|\vec{q}|\le q_D}d^3\vec{q}\right) = \frac{q_D^3}{3}\,\delta_{i,j}. \tag{S4}$$

Substituting Eq. (S4) into Eq. (S3), we obtain

$$\int_0^{q_D}\left\{\frac{S_T(q,\omega)}{k_BT}+\frac{S_L(q,\omega)}{k_BT}\right\}dq = \frac{q_D^3}{2\omega^2}\frac{1}{3N}\sum_{k=1}^{3N}\delta(\omega-\omega_k)\left(\sum_{i=1}^{N}\frac{|\vec{e}_{k,i}|^2}{m_i}\right) = \frac{q_D^3}{2M(\omega)}\frac{g(\omega)}{\omega^2}. \tag{S5}$$

Here, we define the effective mass $M(\omega)$ by $M(\omega)^{-1} = \sum_{i=1}^{N}|\vec{e}_{k,i}|^2/m_i$, evaluated at $\omega = \omega_k$. Thus, $M(\omega)$ depends on the vibrational mode k and hence on the frequency. Equation (S5) corresponds to Eq. (2) in the main paper.

In theoretical analyses, Eq. (2) (or equivalently Eq. (S5)) is used to compute the vDOS from the dynamical structure factor (or from Green's functions). As shown above, Eq. (2) relies on the isotropic-medium assumption in Eq. (S4), which is generally a good approximation in the low-frequency regime.

III. EFFECTIVE-MEDIUM MEAN-FIELD ANALYSIS

Here, we carry out an effective-medium theory (EMT) analysis based on random spring networks, following Refs. [3, 4]. In this framework, particles (nodes) are connected by linear springs to form a random network. Two parameters control stability: the connectivity (contact number) z and the level of pre-stress $e > 0$, which quantifies the internal forces carried by the springs. Mechanical stability requires z to exceed the isostatic threshold $z_c = 2d$ in d dimensions, *i.e.,* a positive excess contact number $\delta z = z - z_c > 0$. In addition, if e exceeds a critical value $e_c > 0$, the network becomes unstable; hence stability requires $e \leq e_c$. At $e = e_c$ the system lies at the stability boundary, *i.e.,* it is marginally stable in this mean-field sense. We stress, however, that this notion of "marginal stability" is defined within the effective-medium (mean-field) framework and does not coincide in all details with the marginal stability of quenched glasses studied in our simulations and experiments, as discussed below. Under the EMT approximation, one can compute the complex, frequency-dependent effective spring constant $k_{\text{eff}}(\omega)$ that characterizes the elastic response of the network. For detailed derivations and the full formulation, see Refs. [3, 4].

Given $k_{\text{eff}}(\omega)$, the vDOS $g(\omega)$ and the dynamical structure factor $S(q,\omega)$ are computed via

$$g(\omega) = \frac{2m\omega}{\pi} \,\text{Im}\left[\frac{3}{q_D^3}\int_0^{q_D} \frac{q^2\,dq}{k_{\text{eff}}(\omega)\,q^2 - m\omega^2}\right], \tag{S6}$$

$$S(q,\omega) = \frac{k_BT}{\pi}\,\frac{q^2}{\omega}\,\text{Im}\left[\frac{1}{k_{\text{eff}}(\omega)\,q^2 - m\omega^2}\right], \tag{S7}$$

where q_D denotes the Debye wavenumber, and Im denotes the imaginary part. In this theory transverse and longitudinal polarizations are not distinguished, so $S(q,\omega) = S_L(q,\omega) = S_T(q,\omega)/2$. From these equations, we obtain

$$\frac{g(\omega)}{\omega^2} = \frac{2m}{q_D^3}\int_0^{q_D} 3\,\frac{S(q,\omega)}{k_BT}\,dq, \tag{S8}$$

which corresponds to Eq. (2) in the main paper upon identifying $3S \to S_T + S_L$ (sum over two transverse and one longitudinal polarizations) and, in the EMT setting with identical particles, m playing the role of the effective mass $M(\omega)$. In what follows, we set the Debye wavenumber to $q_D = 4$ (dimensionless unit of the theory) and $m = 1$.

First, to eliminate pre-stress we set $e = 0$ and tune the connectivity to $z = z_c$. This corresponds to the isostatic-network system. Figure S6 shows $g(\omega)$ in (a) and $g(\omega)/\omega^2$ in (b) (cyan curves). As $\omega \to 0$, $g(\omega)$ remains finite and $g(\omega)/\omega^2 \to \infty$. The low-frequency spectrum at isostaticity comprises zero-frequency floppy modes together with many additional soft, low-frequency modes of isostatic origin.

Next, we introduce excess constraints by setting $\delta z = z - z_c = 10^{-2}$ while keeping $e = 0$. This corresponds to the overconstrained-network system (often termed the "unstressed system" in the effective-medium literature). In this case $g(\omega) \to 0$ as $\omega \to 0$, and $g(\omega)/\omega^2$ converges to the Debye level A_D, *i.e.,* $g(\omega) \simeq A_D\,\omega^2$ (green curves). Introducing excess constraints lifts both the floppy modes and the additional soft modes to finite frequencies, where they merge into a nonphononic band that forms the BP.

Finally, we include pre-stress to mimic internal stresses by setting $e = e_c(1 - 5 \times 10^{-4})$ with $\delta z = 10^{-2}$ held fixed. This choice indicates $1 - e/e_c = 5 \times 10^{-4}$, *i.e.,* the network is tuned arbitrarily close to the marginally stable point. The EMT treats vibrational modes as spatially extended and therefore does not capture localization or the characteristic ω^4 vDOS of QLVs; consequently, the EMT notion of "marginal stability" differs from that realized in quenched glasses in simulations and experiments. In particular, at exactly $e = e_c$ the mean-field BP collapses to $\omega_{\text{BP}} \to 0$, which disagrees with simulations and experiments. To enable a meaningful comparison with the original system in the main paper (quenched glasses studied in our simulations and experiments), we therefore adopt the slightly subcritical choice $e = e_c(1 - 5 \times 10^{-4})$. Under this pre-stress, the isostaticity-derived soft-mode band shifts toward lower frequencies, and the BP correspondingly moves downward as ω_{BP} decreases (purple curves), in agreement with our simulations.

Consistency with our simulations in the main paper is also found in the dynamical structure factor. Figure S7 shows $S(q,\omega)/(k_BT)$ for the original system ($\delta z = 10^{-2}$, $e = e_c(1 - 5 \times 10^{-4})$) in (a) and for the overconstrained-network system ($\delta z = 10^{-2}$, $e = 0$) in (b). At low wavenumbers and frequencies, phonons follow the linear dispersion $\omega = cq$, where c is the sound speed. In addition, a broad, wavenumber-independent (dispersionless) nonphononic band appears around the BP in both cases. Compared with the overconstrained-network case, pre-stress in the original system shifts this band to lower frequencies; this downward shift enhances hybridization between isostaticity-derived soft modes and phonons, thereby broadening the phonon ridge along $\omega = cq$, consistent with our simulation results.

Taken together, these EMT predictions account for, and are consistent with, our simulations and the experimental observations, establishing a coherent picture across theory, simulation, and experiment.

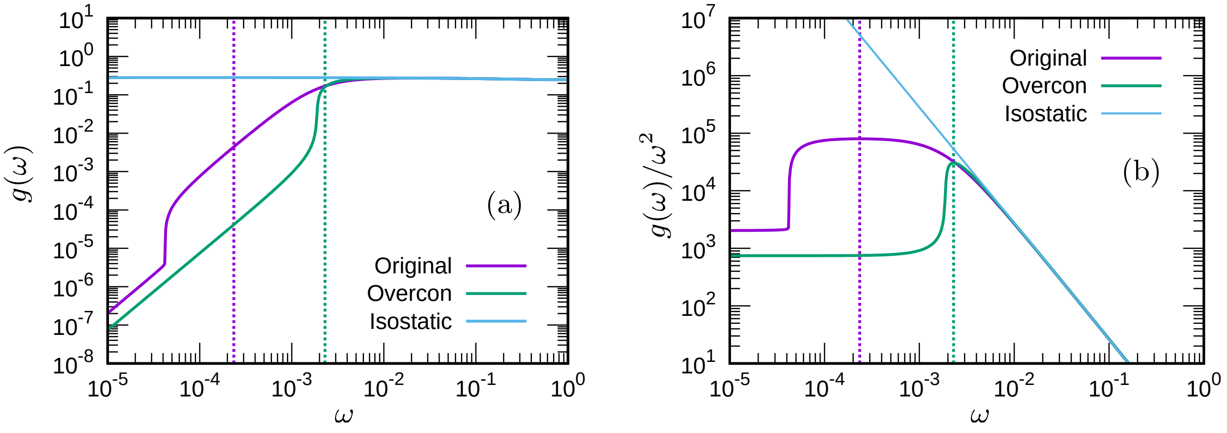

FIG. S6. Vibrational density of states predicted by the effective-medium theory. Panels (a) and (b) show $g(\omega)$ and $g(\omega)/\omega^2$, respectively, for the original system (purple), the overconstrained-network system (green), and the isostatic-network system (cyan). Parameter choices are as follows: the original system has $\delta z = 10^{-2}$ and $e = e_c(1 - 5 \times 10^{-4})$; the overconstrained-network system has $\delta z = 10^{-2}$ and $e = 0$; and the isostatic-network system has $\delta z = 0$ and $e = 0$. The vertical dotted lines mark the BP frequency $\omega_{\rm BP}$ of the original system (purple) and the overconstrained-network system (green). For the isostatic-network system, $\omega_{\rm BP} \to 0$.

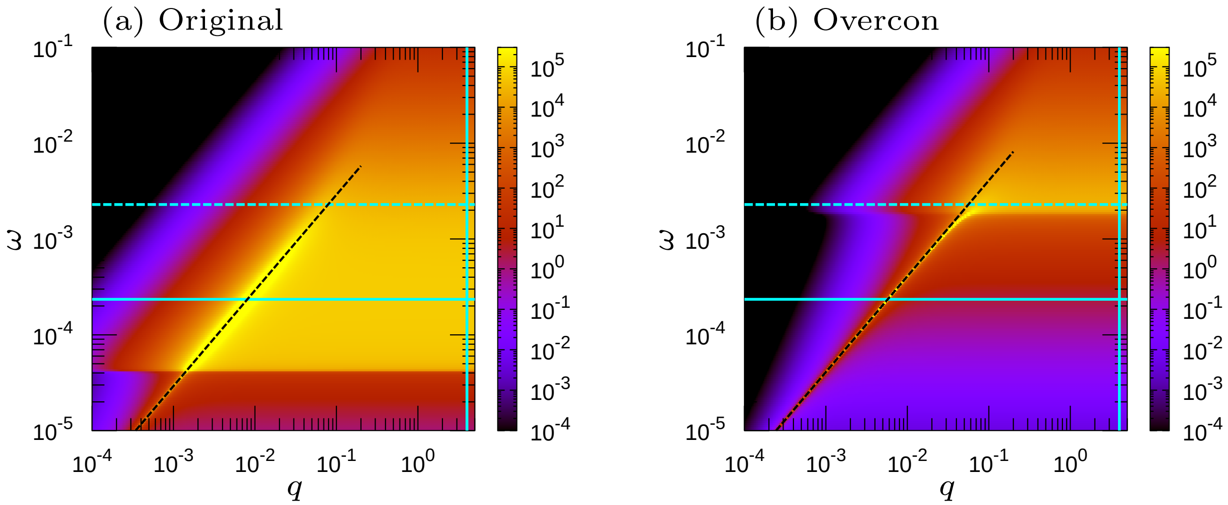

FIG. S7. Dynamical structure factor predicted by the effective-medium theory. Panels (a) and (b) show $S(q,\omega)/(k_B T)$ for the original system and the overconstrained-network system, respectively. Parameter choices: the original system has $\delta z = 10^{-2}$ and $e = e_c(1 - 5 \times 10^{-4})$, and the overconstrained-network system has $\delta z = 10^{-2}$ and $e = 0$. For reference, the vertical line marks the Debye wavenumber q_D, and the horizontal solid and dashed lines indicate the BP frequency $\omega_{\rm BP}$ for the original system and the overconstrained-network system, respectively. The black dashed curve shows the linear dispersion $\omega = cq$, corresponding to phonon excitations.

IV. SILICA GLASS MODELED WITH THE VASHISHTA POTENTIAL

In the main text, we employed the SHIK potential, in which the O–Si–O and Si–O–Si angular constraints are effectively incorporated through suitably parameterized two-body interactions. In this sense, although the potential does not contain explicit three-body terms, the underlying angular rigidity is already built in at the level required to reproduce relevant experimental observables [5]. For this reason, we do not expect that explicitly including three-body interactions would alter the main results or conclusions of this work concerning isostaticity and marginal stability in silica glass.

Nevertheless, it is important to examine this point explicitly by employing a model that includes three-body interactions and by directly comparing its behavior with that obtained using the SHIK potential. Potentials such as the Vashishta model [6], which incorporates explicit angular terms, provide a natural framework for this purpose. Moreover, it is crucial to demonstrate that the new, logically controlled analysis of interatomic interactions in covalent network glasses proposed in the main paper—an analysis that we have applied to the SHIK potential to reveal isostaticity and marginal stability—is broadly applicable to a wide class of models. To this end, here we apply the same analysis to the Vashishta model and examine whether it likewise reveals isostaticity and marginal stability.

A. System description

The Vashishta potential consists of a pairwise (two-body) contribution and an angular (three-body) contribution. Here, we follow the notation used in Ref. [8] and describe the potential as follows.

The two-body interaction is written as

$$v^{(2)}_{\alpha\beta}(r) = \frac{H_{\alpha\beta}}{r^{\eta_{\alpha\beta}}} + \frac{Z_\alpha Z_\beta}{r} \exp\left(-\frac{r}{\lambda_{1,\alpha\beta}}\right) - \frac{D_{\alpha\beta}}{r^4} \exp\left(-\frac{r}{\lambda_{4,\alpha\beta}}\right) - \frac{W_{\alpha\beta}}{r^6}, \tag{S9}$$

where r is the interparticle distance, and α and β denote either Si or O. In Eq. (S9), the first term represents a short-range repulsion, the second term a screened Coulomb interaction, the third term a screened charge–dipole attraction, and the fourth term a van der Waals attraction. The parameters $H_{\alpha\beta}$, $\eta_{\alpha\beta}$, $\lambda_{1,\alpha\beta}$, $D_{\alpha\beta}$, $\lambda_{4,\alpha\beta}$, and $W_{\alpha\beta}$ are specified in Table S2, as reported in Ref. [6]. The charge of silicon is set to $Z_{\mathrm{Si}} = 1.6\,e$ (with e the elementary charge) and, to ensure charge neutrality, the charge of oxygen is set to $Z_{\mathrm{O}} = -Z_{\mathrm{Si}}/2 = -0.8\,e$. The potential $v^{(2)}_{\alpha\beta}(r)$ is truncated at $r = r^{(2)}_c = 10.0$ Å. To prevent discontinuities in the potential and its force (the first derivative of the potential) at the cutoff distance, $v^{(2)}_{\alpha\beta}(r)$ is smoothed as follows:

$$\phi^{(2)}_{\alpha\beta}(r) = v^{(2)}_{\alpha\beta}(r) - v^{(2)}_{\alpha\beta}(r^{(2)}_c) - (r - r^{(2)}_c)\, v^{(2)\prime}_{\alpha\beta}(r^{(2)}_c), \tag{S10}$$

where $\prime$ denotes the first derivative with respect to r. We note that, for the present parameter set reported in Ref. [6] and specified in Table S2, $W_{\alpha\beta} = 0$, so the van der Waals contribution is absent in our implementation.

The three-body interaction is written as

$$v^{(3)}_{\alpha\beta\gamma}(\theta) = B_{\alpha\beta\gamma} \frac{\left(\cos\theta - \cos\theta_{\alpha\beta\gamma}\right)^2}{1 + C_{\alpha\beta\gamma}\left(\cos\theta - \cos\theta_{\alpha\beta\gamma}\right)^2}, \tag{S11}$$

TABLE S2. Parameters for the two-body interaction. $Z_{\mathrm{Si}} = 1.6\,e$, $Z_{\mathrm{O}} = -Z_{\mathrm{Si}}/2 = -0.8\,e$, and $r^{(2)}_c = 10.0$ Å.

α–β	$H_{\alpha\beta}$ (eV Å$^{\eta_{\alpha\beta}}$)	$\eta_{\alpha\beta}$	$\lambda_{1,\alpha\beta}$ (Å)	$D_{\alpha\beta}$ (eV Å^4)	$\lambda_{4,\alpha\beta}$ (Å)	$W_{\alpha\beta}$ (eV Å^6)
Si–O	163.859	9	999	44.2357	4.43	0.0
O–O	743.848	7	999	22.1179	4.43	0.0
Si–Si	0.82023	11	999	0.0	4.43	0.0

TABLE S3. Parameters for the three-body interaction. $r^{(3)}_c = 2.6$ Å and $\gamma_0 = 1.0$ Å.

$\angle\beta$–α–γ	$B_{\alpha\beta\gamma}$ (eV)	$C_{\alpha\beta\gamma}$	$\theta_{\alpha\beta\gamma}$ (°)
O–Si–O	5.0365	0	109.47122
Si–O–Si	20.146	0	140.1

TABLE S4. Physical quantities including elastic moduli and Debye values in silica glass modeled with the Vashishta potential. The quantities reported are the mass density ρ (g/cm^3), elastic moduli K and G (GPa), Poisson's ratio ν, sound speeds c_L and c_T (m/s), wavenumber q (Å^{-1}), Debye level A_D (THz^{-3}), and frequency ω (THz), each expressed in the indicated units. We note that, for the isostatic 2 system, the nonaffine contribution to the shear modulus, $G_N = 141$ GPa, is only slightly smaller than the affine contribution, $G_A = 144$ GPa; the ratio G_N/G_A is 98% (close to 100%), resulting in a small residual shear modulus of $G \simeq 3$ GPa. However, for an isostatic-network system we naturally expect the elastic moduli to vanish. Indeed, for the isostatic-network system constructed for the SHIK potential and for the isostatic 1 system, we confirm that both the bulk modulus and the shear modulus are zero. For this reason, we attribute the small residual value of G and the resulting sound speeds and Debye values (marked by $(*)$) in isostatic 2 to finite-size effects, as observed in jammed systems [7], and related numerical uncertainties, although a more detailed analysis using larger system sizes will be required to fully clarify its origin.

	ρ	K	K_A	K_N	$\frac{K_N}{K_A}$ (%)	G	G_A	G_N	$\frac{G_N}{G_A}$ (%)	ν	c_L	c_T	$\frac{c_L}{c_T}$	q_D	A_D	ω_D	ω_{BP}
Original	2.20	57.7	205	147	71.9	40.3	128	87.5	68.5	0.217	7117	4280	1.66	1.58	0.00179	11.9	1.66
Overcon	2.20	87.4	243	156	64.1	47.5	151	104	68.6	0.270	8280	4648	1.78	1.58	0.00137	13.0	2.59
Isostatic 1	2.20	0	140	140	100	0	88.6	88.6	100	–	0	0	–	1.58	$+\infty$	0	0
Isostatic 2	2.20	0	234	234	100	3 $(*)$	144	141	98 $(*)$	–	$(*)$	$(*)$	–	1.58	$(*)$	$(*)$	0

where α, β, and γ denote either Si or O, and θ is the angle between $\vec{r}_{\alpha\beta} = \vec{r}_\beta - \vec{r}_\alpha$ and $\vec{r}_{\alpha\gamma} = \vec{r}_\gamma - \vec{r}_\alpha$, *i.e.*,

$$\cos\theta = \frac{\vec{r}_{\alpha\beta}\cdot\vec{r}_{\alpha\gamma}}{|\vec{r}_{\alpha\beta}|\,|\vec{r}_{\alpha\gamma}|}. \tag{S12}$$

This term imposes an angular constraint for $\theta = \angle\beta$–α–γ with the α atom as the central atom. The parameters $B_{\alpha\beta\gamma}$, $C_{\alpha\beta\gamma}$, and $\theta_{\alpha\beta\gamma}$ are specified in Table S3, as reported in Ref. [6]. The model includes two types of angular constraints, corresponding to $\theta = \angle$O–Si–O and $\theta = \angle$Si–O–Si. The three-body interaction $v^{(3)}_{\alpha\beta\gamma}(\theta)$ is smoothly truncated for $|\vec{r}_{\alpha\beta}| < r_c^{(3)} = 2.6$ Å and $|\vec{r}_{\alpha\gamma}| < r_c^{(3)} = 2.6$ Å by introducing the smoothing functions $s_{\alpha\beta}(r)$ and $s_{\alpha\gamma}(r)$:

$$\phi^{(3)}_{\alpha\beta\gamma}(\theta) = v^{(3)}_{\alpha\beta\gamma}(\theta)\, s_{\alpha\beta}\big(|\vec{r}_{\alpha\beta}|\big)\, s_{\alpha\gamma}\big(|\vec{r}_{\alpha\gamma}|\big), \tag{S13}$$

with

$$s_{\alpha\beta}(r) = \exp\left(\frac{\gamma_0}{r - r_c^{(3)}}\right), \tag{S14}$$

where $\gamma_0 = 1.0$ Å. We note that $\theta_{\alpha\beta\gamma}$ in Eq. (S11) defines the preferred bond angle for the $\angle\beta$–α–γ constraint. For the present parameter set reported in Ref. [6] and specified in Table S3, $C_{\alpha\beta\gamma} = 0$, and Eq. (S11) reduces to a simple quadratic penalty, $v^{(3)}_{\alpha\beta\gamma}(\theta) = B_{\alpha\beta\gamma}\big(\cos\theta - \cos\theta_{\alpha\beta\gamma}\big)^2$.

We performed MD simulations with LAMMPS [9]. Following the same protocol as for the SHIK potential in the main paper, we constructed a silica-glass configuration composed of N atoms (N_{Si} silicon atoms and $N_{\mathrm{O}} = 2N_{\mathrm{Si}}$ oxygen atoms, so that $N = N_{\mathrm{Si}} + N_{\mathrm{O}} = 3N_{\mathrm{Si}}$). The mass density was fixed at $\rho = 2.20$ g/cm^3. Initial Si and O positions were randomized, and the system was equilibrated at $T = 3500$ K for 100 ps to obtain a homogeneous liquid. The system was then cooled to $T = 300$ K at a rate of 1 K/ps and equilibrated at $T = 300$ K for 100 ps. Finally, all atomic velocities were set to zero and the configuration was relaxed by energy minimization to obtain the inherent structure $\vec{r} = [\vec{r}_1, \vec{r}_2, \ldots, \vec{r}_N]$, *i.e.*, $T = 0$ K configuration.

Using this inherent structure $\vec{r} = [\vec{r}_1, \vec{r}_2, \ldots, \vec{r}_N]$, we performed a standard normal-mode analysis by solving the eigenvalue problem of the dynamical matrix (a $3N \times 3N$ matrix) to obtain the eigenvalues λ_k and eigenvectors $\vec{e}_k = [\vec{e}_{k,1}, \vec{e}_{k,2}, \cdots, \vec{e}_{k,N}]$ for modes $k = 1, 2, \cdots, 3N$. From these vibrational modes, we computed $g(\omega)$ and $S_\alpha(q,\omega)$ using Eqs. (11) and (16) of the main paper, respectively. To cover a broad range of wavenumbers q and frequencies ω, we used several system sizes ranging from $N = 1.5\times10^4$ to 6.0×10^4 atoms. For each system size, we performed three independent MD simulations to obtain three inherent-structure configurations. All reported quantities are averaged over these three independent configurations.

As in the main paper for silica glass modeled with the SHIK potential, we calculate the elastic moduli—the bulk modulus K and the shear modulus G—using the harmonic formulation based on linear-response theory [2, 10]. Because the Vashishta potential includes explicit three-body interactions, we employ the corresponding formulation

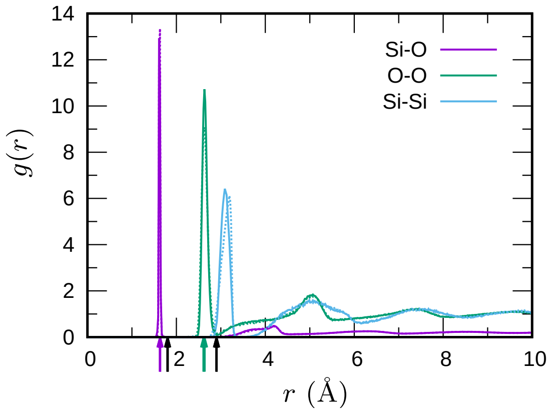

FIG. S8. Comparison of the radial distribution function calculated using the Vashishta and SHIK potentials. The radial distribution function $g(r)$ is plotted as a function of distance r for Si–O (purple), O–O (green), and Si–Si (cyan) pairs. The solid and dotted curves show the results for the Vashishta and SHIK potentials, respectively, which are in almost perfect agreement. For clarity, the Si–O curve is scaled by a factor of 0.2 (*i.e.*, $0.2\,g(r)$). The purple and green arrows mark the first peaks at $r \approx 1.6$ Å (Si–O) and $r \approx 2.6$ Å (O–O), which indicate the typical nearest-neighbor separations. The black arrows indicate the distance cutoffs used to define connectivity: $r \leq 1.8$ Å for Si–O bonds and $r \leq 2.9$ Å for O–O contacts, chosen as the first minima following these peaks. The data for the SHIK potential are the same as those used in the main text.

that accounts for many-body terms [11]. From these moduli, we compute the sound speeds and the Debye quantities, including the Debye frequency ω_D and the Debye level A_D. Table S4 summarizes these elastic moduli, sound speeds, and Debye quantities for the original atomistic system as well as the overconstrained-network and isostatic-network systems.

B. Comparison of static structure and the number of Si–O bonds and O–O contacts in the inherent structure with the SHIK potential

Figure S8 compares the radial distribution function $g(r)$ of the inherent structure obtained with the Vashishta potential with the corresponding data for the SHIK potential. As can be seen, the Vashishta results (solid curves) are in almost perfect agreement with the SHIK results (dotted curves), indicating that, at the level of two-body correlations, the two potentials generate essentially the same inherent structure. In particular, the Si and O atoms form a tetrahedral network with typical nearest-neighbor separations of 1.6 Å for Si–O bonds and 2.6 Å for O–O contacts. Accordingly, following the same definitions as in the main paper for the SHIK system, we define Si–O bonds as Si–O pairs within the cutoff 1.8 Å and O–O contacts as O–O pairs within the cutoff 2.9 Å. Therefore, as we do below, we construct isostatic and overconstrained networks for the Vashishta system using the same bond and contact criteria as for the SHIK system.

However, there is a small difference between the Vashishta and SHIK systems in the number of Si–O bonds and O–O contacts. Using the common definitions of Si–O bonds and O–O contacts given above, the SHIK system has $4.00\,N_{\mathrm{Si}}$ Si–O bonds and $3.00\,N_{\mathrm{O}}$ O–O contacts (Table 1 of the main paper), whereas the Vashishta system has $3.99\,N_{\mathrm{Si}}$ Si–O bonds and $2.99\,N_{\mathrm{O}}$ O–O contacts, *i.e.*, values that are slightly smaller. Importantly, while in the SHIK system all Si atoms form four Si–O bonds, in the Vashishta system about 99% of Si atoms form four Si–O bonds, whereas the remaining 1% form only three Si–O bonds. (Accordingly, in the Vashishta system most O atoms form two Si–O bonds, whereas a small fraction form only one Si–O bond.) As we show below, this small fraction of undercoordinated Si atoms leads to the appearance of several localized vibrational modes in the isostatic-network and overconstrained-network systems. Overall, despite these small differences, the Vashishta and SHIK systems exhibit essentially the same bond and contact statistics.

C. Comparison of vDOS and dynamical structure factor with the SHIK potential

Before analyzing isostaticity and marginal stability for the Vashishta potential, we compare the vDOS and the dynamical structure factor with those obtained using the SHIK potential in Figs. S9 and S10. As seen in Fig. S9, although there are some quantitative differences—for example, the BP frequencies are $\omega_{\mathrm{BP}} = 1.66$ THz and 1.21 THz

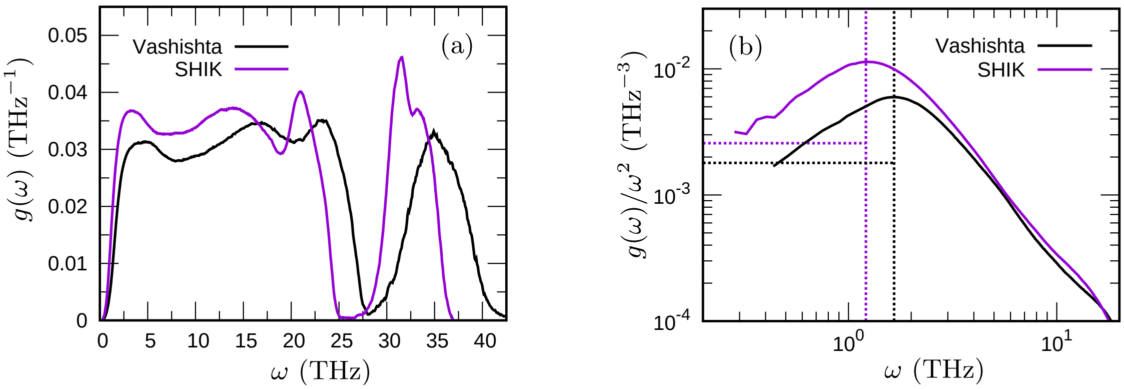

FIG. S9. Comparison of the vDOS calculated using the Vashishta and SHIK potentials. Panels (a) and (b) respectively show $g(\omega)$ and $g(\omega)/\omega^2$ for the Vashishta (black) and SHIK (purple) potentials. The vertical dotted lines in (b) indicate the BP frequencies $\omega_{\rm BP} = 1.66$ THz and 1.21 THz for the Vashishta and SHIK potentials, respectively. The horizontal dotted lines in (b) indicate the Debye level A_D. The data for the SHIK potential are the same as those used in the main text.

for the Vashishta and SHIK potentials, respectively—the overall shape of the vDOS is in qualitative agreement between the two. We also note that, when compared with experimental vDOS data, the SHIK potential provides a more accurate quantitative description than the Vashishta potential.

Furthermore, in the dynamical structure factor presented in Fig. S10, for both potentials we observe phonon excitations at low frequencies ω and low wavenumbers q along the linear dispersion curves, as well as a broad, approximately wavenumber-independent band emerging around and above the BP and extending up to q_D, corresponding to non-phononic excitations. These features are consistent with inelastic X-ray scattering experiments. The good agreement between the Vashishta- and SHIK-based results suggests that adding explicit three-body interactions does not alter the main results and conclusions of this work.

In the next section, we apply to the Vashishta potential the same controlled analysis of interatomic interactions that we used for the SHIK potential in the main paper, and examine isostaticity and marginal stability.

D. Two nearly isostatic networks constructed in the Vashishta system

For the SHIK system, we constructed an isostatic-network system; using the same procedure, we can also construct an isostatic-network system for the Vashishta system. The key point is to consider only the Si–O bonds. To this end, for the two-body part, we connect Si–O bonds, *i.e.*, Si–O pairs within a cutoff distance of 1.8 Å, by unstressed (neither pre-stretched nor pre-compressed) springs. The spring constant is taken as the second derivative $\phi^{(2)\prime\prime}_{\rm SiO}$ evaluated from the sum of the short-range repulsion and the screened charge–dipole attraction. We note that, in the SHIK potential, the short-range potential $\phi_S(r)$ effectively corresponds to the sum of the short-range repulsion, the screened charge–dipole attraction, and the van der Waals attraction in the Vashishta form. As noted above, the van der Waals attraction is absent in the present parameter set; therefore, to enable a direct comparison with the SHIK case, we consider the sum of the short-range repulsion and the screened charge–dipole attraction, and we do not include any contribution from the screened Coulomb interaction.

For the three-body part, we consider only the ∠O–Si–O angular constraint associated with the SiO_4 tetrahedra, and attach an unstressed spring to each ∠O–Si–O angle with spring constant given by the second derivative $\phi^{(3)\prime\prime}_{\rm SiOO}$. We do not include the ∠Si–O–Si angular constraint. With these choices, the resulting network becomes *nearly* isostatic, in the sense that the number of degrees of freedom $N_{\rm dof}$ *nearly* equals the number of constraints $N_{\rm const}$.

Here, *nearly* means that, while the SHIK network is strictly isostatic with $N_{\rm const} = N_{\rm dof}$, the Vashishta network has a slightly smaller number of constraints than degrees of freedom due to a small fraction of undercoordinated Si atoms. As explained above, in the Vashishta system about 99% of Si atoms form four Si–O bonds, whereas the remaining 1% form only three Si–O bonds. (A small fraction of O atoms also deviates from twofold coordination, forming only one Si–O bond; however, we neglect this effect because it is much smaller than that of the undercoordinated Si atoms.) Taking this into account, Eq. (1) of the main paper yields

$$N_{\rm const} = N_{\rm O}\cdot\frac{2}{2} + 0.99\,N_{\rm Si}\cdot(2+5) + 0.01\,N_{\rm Si}\cdot\left(\frac{3}{2}+3\right) \approx 8.98\,N_{\rm Si} \lesssim 9\,N_{\rm Si} = N_{\rm dof}. \tag{S15}$$

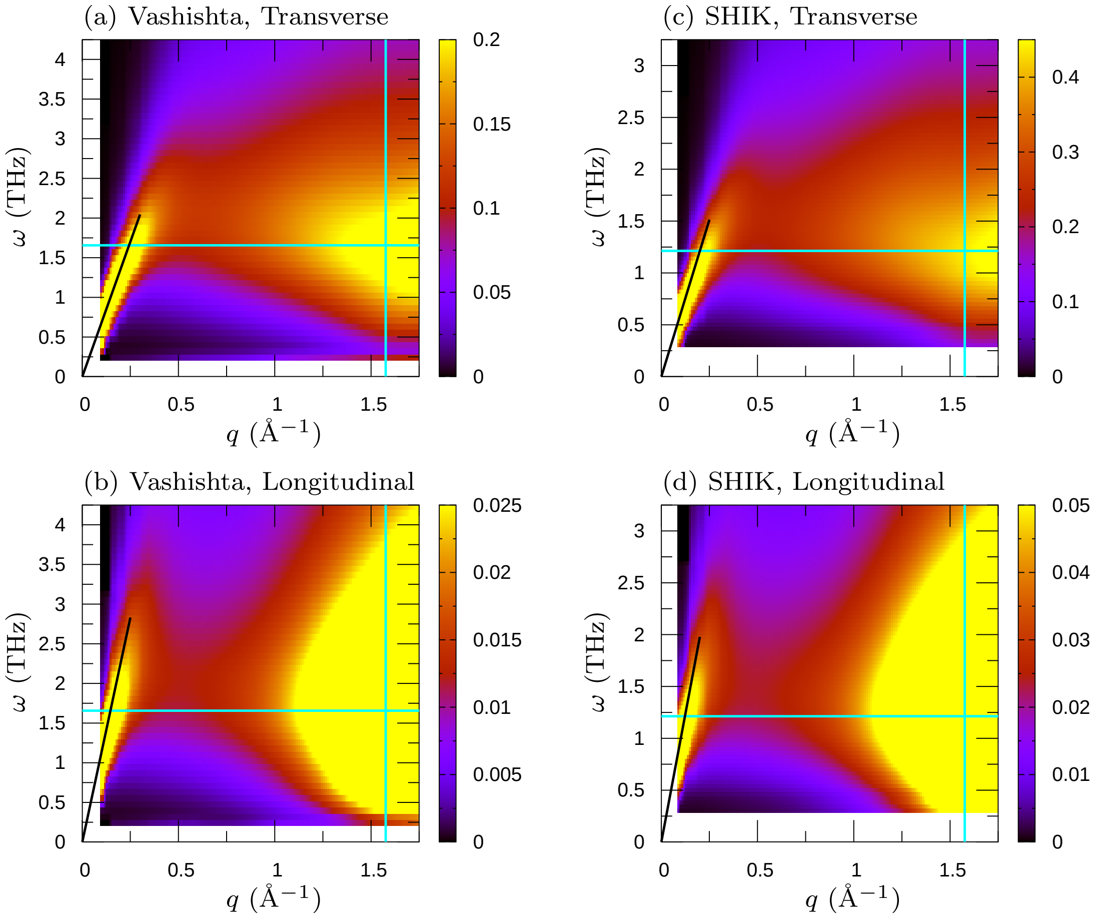

FIG. S10. Comparison of the dynamical structure factor calculated using the Vashishta and SHIK potentials. Panels (a) and (b) show the transverse and longitudinal dynamical structure factors, $S_T(q,\omega)/(k_BT)$ and $S_L(q,\omega)/(k_BT)$, for the Vashishta potential, while panels (c) and (d) show $S_T(q,\omega)/(k_BT)$ and $S_L(q,\omega)/(k_BT)$ for the SHIK potential. Values are reported in units of $(\mathrm{eV\,THz})^{-1}$. The vertical line marks the Debye wavenumber q_D, and the horizontal solid line indicates the BP frequency ω_{BP}. The black solid curve shows the linear dispersion $\omega = c_T q$ in (a,c) or $\omega = c_L q$ in (b,d), where c_T and c_L are the transverse and longitudinal sound speeds, respectively, corresponding to phonon excitations. The data for the SHIK potential are the same as those used in the main text.

Thus, $N_{\mathrm{dof}} - N_{\mathrm{const}} \approx 0.02\,N_{\mathrm{Si}}$, implying the appearance of a small fraction of zero-energy-cost modes. Specifically, the fraction is $0.02\,N_{\mathrm{Si}}/(3N) \approx 0.0022$, *i.e.*, 0.22% of all vibrational modes. For $N = 1.5\times10^4$ atoms (*i.e.*, $3N = 45{,}000$ modes in total), this corresponds to 100 zero-energy-cost modes. In what follows, we treat this *nearly* isostatic network as an isostatic-network system and refer to it as *isostatic 1*.

In addition, in contrast to the SHIK system, for the Vashishta system we can construct another, distinct isostatic network. Starting from *isostatic 1*, we additionally connect O–O contacts, *i.e.*, O–O pairs within a cutoff distance of 2.9 Å, by unstressed springs with spring constants given by the second derivative $\phi_{\mathrm{OO}}^{(2)\prime\prime}$ evaluated from the sum of the short-range repulsion and the screened charge–dipole attraction. The point here is that the constraints introduced by these O–O springs are not independent of the ∠O–Si–O angular constraints; consequently, the number of independent constraints does not increase and is the same as in *isostatic 1*. Therefore, the resulting network also remains *nearly* isostatic, with $N_{\mathrm{dof}} - N_{\mathrm{const}} \approx 0.02\,N_{\mathrm{Si}}$. We refer to this second isostatic network as *isostatic 2*. For the SHIK system, the effect of the O–Si–O angular constraint is effectively incorporated through suitably parameterized two-body O–O interactions; thus, the isostatic network constructed for SHIK in the main paper corresponds to *isostatic 2* in the Vashishta case, rather than *isostatic 1*.

Figure S11 compares the vDOSs of the two isostatic-network systems, *isostatic 1* and *isostatic 2*. We first note that, as discussed above, both *isostatic 1* and *isostatic 2* have a slightly smaller number of constraints than degrees of freedom due to a small fraction of undercoordinated Si atoms, which results in a small number of zero-frequency modes (about 0.22% of all vibrational modes). After removing these zero-frequency modes, we compute the vDOS and show the results in Fig. S11. As can be seen, for both networks the vDOS remains finite as $\omega \to 0$, indicating

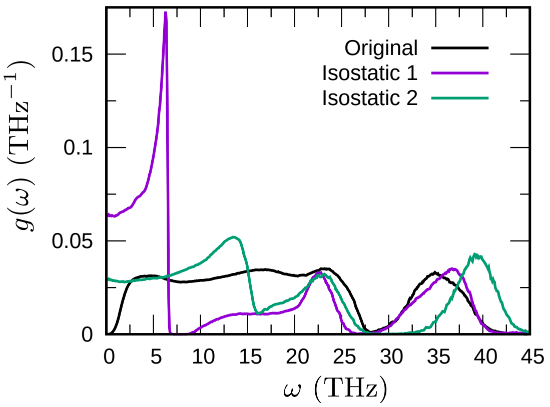

FIG. S11. The vDOS in the isostatic-network systems calculated using the Vashishta potential. The vDOS $g(\omega)$ is plotted as a function of frequency ω for two isostatic-network systems, *isostatic 1* (purple) and *isostatic 2* (green). We also show the result for the original atomistic system (black) for reference.

a gapless spectrum that comprises soft, low-frequency modes of isostatic origin. Compared to *isostatic 2*, *isostatic 1* exhibits a larger number of low-frequency soft modes because it lacks springs between O–O contacts. Although the vDOS differs between the two networks, the key result is the same: both are (nearly) isostatic and exhibit a finite vDOS as $\omega \to 0$. We note again that *isostatic 2* corresponds to the isostatic network constructed for SHIK in the main paper, and that its vDOS is closer in shape to the SHIK isostatic result shown in Fig. 2 of the main paper.

In what follows, by discussing the Vashishta results in comparison with the SHIK results, we demonstrate that the method developed in the main paper to reveal isostaticity and marginal stability for the SHIK potential is also applicable to the Vashishta potential, and that the main results and conclusions for silica glass obtained from the SHIK potential remain unchanged. For this purpose, we focus on *isostatic 2*, which corresponds to the SHIK isostatic network.

E. Isostaticity and marginal stability

Figure S12 focuses on the low-frequency regime and presents the vDOS $g(\omega)$, the reduced vDOS $g(\omega)/\omega^2$, and the participation ratio $\mathcal{P}_k$ as functions of frequency ω. For comparison, the corresponding results for the SHIK potential are shown alongside. For the isostatic-network system (cyan), as discussed above, $g(\omega)$ remains finite as $\omega \to 0$; consequently, $g(\omega)/\omega^2$ diverges toward the zero-frequency limit, indicating that the BP enhancement persists and grows as $\omega \to 0$. This behavior is the same as that observed for the SHIK system.

In contrast, the participation-ratio data indicate that several soft modes associated with isostaticity are spatially localized in the Vashishta case. This localization is not caused directly by the distribution of spring constants in the Vashishta potential; rather, it originates from the presence of a small fraction of undercoordinated Si atoms in the network generated by the Vashishta potential. Indeed, in Fig. S14 we show results for an isostatic network built from the inherent structure generated with the SHIK potential, but with the two- and three-body spring constants defined from the Vashishta potential; in this case, no localization of the isostaticity-induced soft modes is observed. Therefore, we attribute the localization to the fact that about 1% of Si atoms form only three Si–O bonds, so that vibrations are amplified around these undercoordinated (and thus locally weaker) regions. We emphasize again that this is not a direct consequence of the Vashishta potential itself, but rather reflects the structural and bond statistics of the silica glass it generates. Accordingly, the number of modes that exhibit localization is on the order of $N_{\mathrm{dof}} - N_{\mathrm{const}} \approx 0.02\, N_{\mathrm{Si}}$, while the vast majority of other soft modes remain spatially extended, as in the SHIK case, consistent with our overall picture.

Next, building on the isostatic-network system and analogous to the construction of an overconstrained network for the SHIK system in the main paper, we construct the corresponding overconstrained network for the Vashishta system as follows. For the two-body part, in addition to the Si–O pairs within a cutoff distance of 1.8 Å included in the isostatic-network system, we connect all O–O pairs within the potential cutoff distance $r_c^{(2)} = 10.0$ Å by unstressed springs, with spring constants given by the second derivative $\phi_{\mathrm{OO}}^{(2)\prime\prime}$ evaluated from the sum of the short-range repulsion and the screened charge–dipole attraction. For the three-body part, in addition to the ∠O–Si–O angular constraint, we also include the ∠Si–O–Si angular constraint, and attach an unstressed spring to each ∠Si–O–Si angle with spring constant given by the second derivative $\phi_{\mathrm{OSiSi}}^{(3)\prime\prime}$. In this case, the system is overconstrained, $N_{\mathrm{const}} > N_{\mathrm{dof}}$, and the

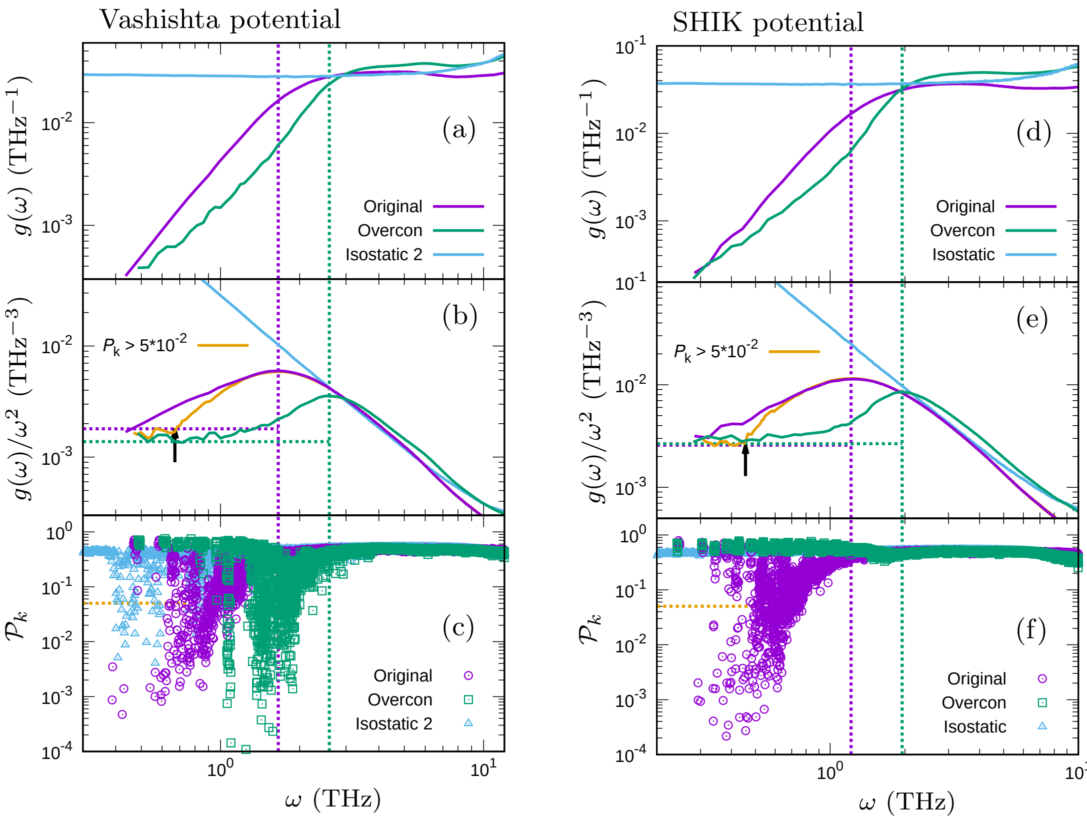

FIG. S12. Vibrational states in the low-frequency regime calculated using the Vashishta and SHIK potentials. Panels (a–c) show results for the Vashishta potential, and panels (d–f) show those for the SHIK potential. $g(\omega)$, $g(\omega)/\omega^2$, and $\mathcal{P}_k$ are plotted as functions of frequency ω for the original system (purple), the overconstrained-network system (green), and the isostatic-network system (cyan). The vertical lines mark the BP frequency ω_{BP} for the original and overconstrained-network systems, while the horizontal dotted lines in (b,e) indicate the Debye level A_D. In addition, panels (b,e) show, in orange, the vDOS $g_{\mathrm{EXT}}(\omega)$ of extended modes with $\mathcal{P}_k > \mathcal{P}_{\mathrm{th}} = 5 \times 10^{-2}$. This threshold $\mathcal{P}_{\mathrm{th}}$ is indicated by the horizontal dotted line in (c,f). The arrow in (b,e) marks the continuum-limit frequency ω_0 at which $g_{\mathrm{EXT}}(\omega)/\omega^2$ converges to A_D. The vDOS of QLVs, $g_{\mathrm{QLV}}(\omega)$, corresponding to modes with $\mathcal{P}_k \leq \mathcal{P}_{\mathrm{th}}$, is shown in Fig. S13. The data for the SHIK potential are the same as those used in the main text.

vDOS vanishes as $\omega \to 0$, as in the SHIK case.

Focusing on the overconstrained-network system (green) in Fig. S12, $g(\omega)/\omega^2$ displays a clear BP. Below the BP frequency ω_{BP}, $g(\omega)/\omega^2$ converges to the Debye level A_D, hence $g(\omega) \to A_D\omega^2$, and the vibrational states are extended phonons with large $\mathcal{P}_k$. Above ω_{BP}, the soft modes of isostatic origin (zero-frequency floppy modes and additional nonzero-frequency modes) are lifted to finite frequencies and merge into a nonphononic band; their accumulation produces the excess over the Debye law, *i.e.*, the BP. These behaviors are again fully consistent with the SHIK results. Unlike the SHIK case, however, several soft modes remain spatially localized even in the overconstrained-network system, reflecting the localization already present in the isostatic-network system. As shown in Fig. S14, this localization does not occur when we use an inherent structure generated with the SHIK potential; thus, it is not a direct consequence of the Vashishta potential itself, but rather reflects the structural and bond statistics of the silica glass it generates. Nevertheless, the vast majority of other soft modes remain spatially extended, as in the SHIK case, consistent with our overall picture.

Finally, to move from the overconstrained-network system to the original atomistic system, we reinstate all effects neglected in constructing the overconstrained network, including contributions from the screened Coulomb interaction as well as the internal stresses introduced by replacing unstressed springs with stressed ones. With these ingredients restored, the isostaticity-derived band of soft modes shifts toward lower frequencies, and the BP accordingly moves downward as ω_{BP} decreases (purple) in Fig. S12. Notably, QLVs with low $\mathcal{P}_k$ emerge at the low-ω edge below ω_{BP}. We partition modes into extended modes with $\mathcal{P}_k > \mathcal{P}_{\mathrm{th}}$ and QLVs with $\mathcal{P}_k \leq \mathcal{P}_{\mathrm{th}}$, and compute their vDOSs, $g_{\mathrm{EXT}}(\omega)$ and $g_{\mathrm{QLV}}(\omega)$, separately. Here we take $\mathcal{P}_{\mathrm{th}} = 5 \times 10^{-2}$, as in the SHIK case. We observe that $g_{\mathrm{EXT}}(\omega)$ converges to the Debye law $A_D\omega^2$ at a characteristic frequency ω_0 (orange curve in panel (b) of Fig. S12). Furthermore, we find that $g_{\mathrm{QLV}}(\omega)$ follows the ω^4 scaling law, $g_{\mathrm{QLV}}(\omega) \propto \omega^4$, as shown in Fig. S13. Importantly, the QLVs exhibit a gapless

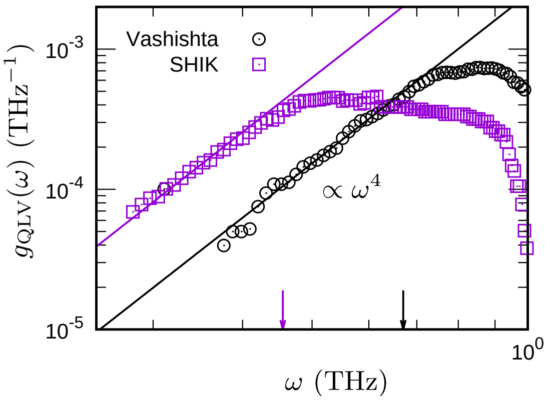

FIG. S13. Vibrational density of states of quasi-localized vibrations (QLVs). The vDOS of QLVs, $g_{\rm QLV}(\omega)$, is plotted as a function of frequency ω for the Vashishta (black circles) and SHIK (purple squares) potentials. The solid lines indicate the scaling $g_{\rm QLV}(\omega) \propto \omega^4$. The arrows mark the continuum-limit frequency ω_0 at which $g_{\rm EXT}(\omega)/\omega^2$ converges to A_D, as shown in Fig. S12. The data for the SHIK potential are the same as those used in the main text.

vDOS with a power-law dependence on ω, indicating that silica glass is driven into a marginally stable state. These behaviors obtained with the Vashishta potential are fully consistent with those obtained with the SHIK potential.

F. Dynamical structure factor

Figure S15 shows the transverse dynamical structure factor $S_T(q,\omega)$, focusing on the low-frequency and low-wavenumber ($q \lesssim q_D$) regime. The figure includes data for both the original system and the overconstrained-network system. For comparison, the corresponding results for the SHIK potential are also shown.

Looking at the overconstrained-network system in panel (b) of Fig. S15, phonon excitations appear at low ω and low q along the linear dispersion relation $\omega = c_T q$, where c_T is the transverse sound speed. In addition, a broad, approximately wavenumber-independent band emerges around and above the BP and extends up to q_D, indicating nonphononic excitations. This band originates from isostaticity-derived modes that, in the presence of excess constraints, are lifted to finite frequencies. Therefore, the BP is built from two components: linearly dispersing phonons that follow $\omega = c_T q$ and a nearly dispersionless band of isostaticity-derived modes. These results are fully consistent with those for the SHIK system.

Turning to the original atomistic system in panel (a), restoring the internal stresses introduced by replacing unstressed springs with stressed ones shifts the entire nonphononic band to lower frequencies, and $\omega_{\rm BP}$ decreases accordingly. At the low-ω edge below $\omega_{\rm BP}$, a band of QLV excitations appears. Phonon ridges along the linear dispersion $\omega = c_T q$ remain visible, but they are broader than in the overconstrained-network system. This broadening is attributed to isostaticity-derived modes shifting into the low-frequency range and hybridizing with the phonons, thereby increasing the phonon linewidth. In this way, marginal stability manifests as a wavenumber-independent band that is shifted downward by internal stresses, together with the emergence of QLV excitations at the low-frequency edge. Again, these results are fully consistent with those for the SHIK system.

G. Conclusion for silica glass modeled with the Vashishta potential

In this section, we analyzed silica glass modeled with the Vashishta potential. Specifically, we applied to the Vashishta system the new, logically controlled analysis of interatomic interactions in covalent network glasses proposed in the main paper—an analysis that we previously applied to the SHIK potential to reveal isostaticity and marginal stability—and demonstrated that the method is broadly applicable beyond a specific potential model. As a result, we found that the main results and conclusions regarding isostaticity and marginal stability in silica glass, obtained for the SHIK system in the main paper, are also reproduced with the Vashishta potential. This agreement suggests that our conclusions are robust and are expected to hold independently of the particular potential model used. In particular, our Vashishta-based analysis indicates that explicitly including three-body interactions does not alter the main results or conclusions of this work concerning isostaticity and marginal stability in silica glass.

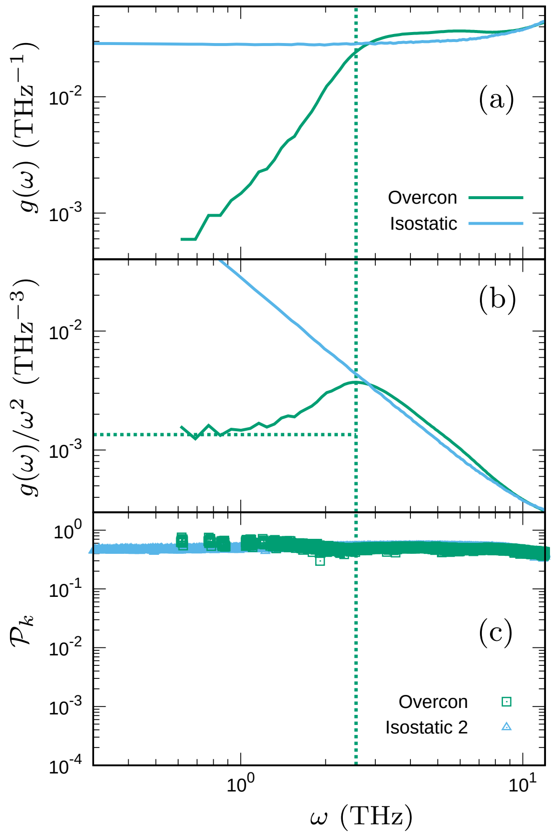

FIG. S14. Vibrational states in the low-frequency regime calculated using the Vashishta potential on the inherent structure generated with the SHIK potential. (a) $g(\omega)$, (b) $g(\omega)/\omega^2$, and (c) $\mathcal{P}_k$ are plotted as functions of frequency ω for the overconstrained-network system (green) and the isostatic-network system (cyan). The vertical dotted line marks the BP frequency $\omega_{\rm BP}$ for the overconstrained-network system, whereas the horizontal dotted line in (b) indicates the Debye level A_D for the overconstrained-network system. We note that, when we construct the overconstrained and isostatic networks from the inherent structure generated with the SHIK potential and then perform the vibrational analysis using the Vashishta potential, this procedure is natural because the forces vanish in these network systems and force balance is satisfied. By contrast, for the original atomistic system, the same procedure would be inappropriate because the inherent structure generated with the SHIK potential is not force-balanced under the Vashishta potential and therefore carries finite residual forces.

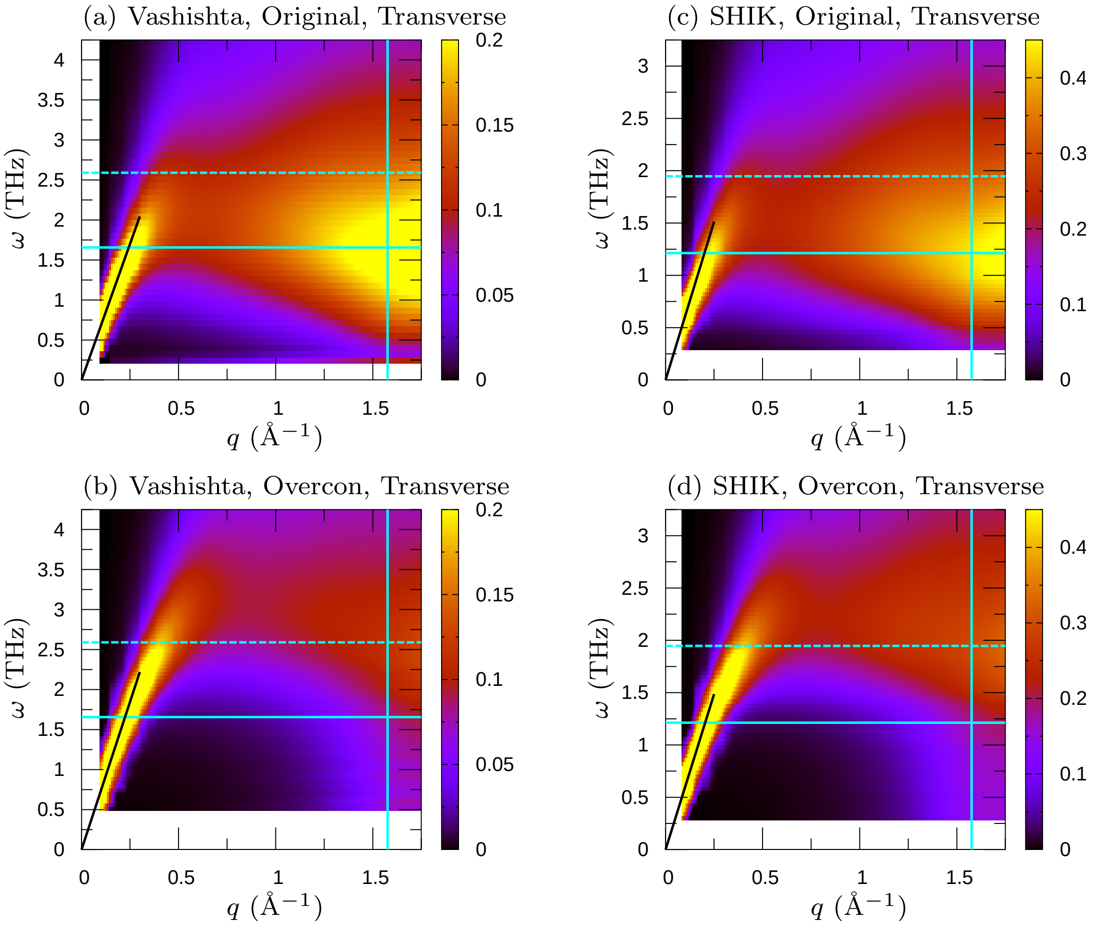

FIG. S15. Transverse dynamical structure factor calculated using the Vashishta and SHIK potentials. (a,b) Vashishta potential and (c,d) SHIK potential. $S_T(q,\omega)/(k_B T)$ is shown as a function of q and ω for the original systems in (a,c) and the overconstrained-network systems in (b,d). Values are reported in units of $(\mathrm{eV\,THz})^{-1}$. The vertical line marks the Debye wavenumber q_D. Horizontal solid and dotted lines indicate the BP frequency ω_{BP} for the original and overconstrained-network systems, respectively. The black solid curve shows the linear dispersion $\omega = c_T q$, with c_T being the transverse sound speed, corresponding to phonon excitations. The data for the SHIK potential are the same as those used in the main text.

[1] G. S. Grest, S. R. Nagel, and A. Rahman, Longitudinal and transverse excitations in a glass, Phys. Rev. Lett. **49**, 1271 (1982).
[2] H. Mizuno and A. Ikeda, Computational Simulations of the Vibrational Properties of Glasses, in *Low-Temperature Thermal and Vibrational Properties of Disordered Solids*, edited by M. A. Ramos (WORLD SCIENTIFIC (EUROPE), 2022) Chap. 10, pp. 375–433.
[3] M. Wyart, Scaling of phononic transport with connectivity in amorphous solids, EPL (Europhysics Letters) **89**, 64001 (2010).
[4] E. DeGiuli, A. Laversanne-Finot, G. Düring, E. Lerner, and M. Wyart, Effects of coordination and pressure on sound attenuation, boson peak and elasticity in amorphous solids, Soft Matter **10**, 5628 (2014).
[5] S. Sundararaman, L. Huang, S. Ispas, and W. Kob, New optimization scheme to obtain interaction potentials for oxide glasses, The Journal of Chemical Physics **148**, 194504 (2018).
[6] P. Vashishta, R. K. Kalia, J. P. Rino, and I. Ebbsjö, Interaction potential for sio2: A molecular-dynamics study of structural correlations, Physical Review B **41**, 12197 (1990).
[7] C. P. Goodrich, S. Dagois-Bohy, B. P. Tighe, M. van Hecke, A. J. Liu, and S. R. Nagel, Jamming in finite systems: Stability, anisotropy, fluctuations, and scaling, Phys. Rev. E **90**, 022138 (2014).
[8] P. S. Branicio, J. P. Rino, C. K. Gan, and H. Tsuzuki, Interaction potential for indium phosphide: a molecular dynamics and first-principles study of the elastic constants, generalized stacking fault and surface energies, Journal of Physics: Condensed Matter **21**, 095002 (2009).
[9] S. Plimpton, Fast parallel algorithms for short-range molecular dynamics, Journal of Computational Physics **117**, 1 (1995).
[10] A. Lemaître and C. Maloney, Sum rules for the quasi-static and visco-elastic response of disordered solids at zero temperature, Journal of Statistical Physics **123**, 415 (2006).
[11] J. Grießer, L. Frérot, J. A. Oldenstaedt, M. H. Müser, and L. Pastewka, Analytic elastic coefficients in molecular calculations: Finite strain, nonaffine displacements, and many-body interatomic potentials, Phys. Rev. Mater. **7**, 073603 (2023).
